# Supplementary material for: Efficacy of dopamine, epinephrine and blood transfusion for treatment of fluid refractory shock in children with severe acute malnutrition or severe underweight and cholera or other dehydrating diarrhoeas: protocol for a randomised controlled clinical trial
Source: BMJ Open. 2023 Apr 11;13(4):e068660. doi: 10.1136/bmjopen-2022-068660 (PMC10106066; doi:10.1136/bmjopen-2022-068660)
Supplement: Author's manuscript [file bmjopen-2022-068660.draft_revisions.pdf]

BMJ Open is committed to open peer review. As part of this commitment we make the peer review history of every article we publish publicly available.

When an article is published we post the peer reviewers' comments and the authors' responses online. We also post the versions of the paper that were used during peer review. These are the versions that the peer review comments apply to.

The versions of the paper that follow are the versions that were submitted during the peer review process. They are not the versions of record or the final published versions. They should not be cited or distributed as the published version of this manuscript.

BMJ Open is an open access journal and the full, final, typeset and author-corrected version of record of the manuscript is available on our site with no access controls, subscription charges or pay-per-view fees (http://bmjopen.bmj.com).

If you have any questions on BMJ Open's open peer review process please email info.bmjopen@bmj.com

BMJ Open

Efficacy of dopamine, adrenaline and blood transfusion for treatment of fluid refractory shock in children with severe acute malnutrition or severe underweight and cholera or other dehydrating diarrheas: protocol for a randomized controlled clinical trial

Journal:	*BMJ Open*
Manuscript ID	bmjopen-2022-068660
Article Type:	Protocol
Date Submitted by the Author:	01-Oct-2022

Complete List of Authors:	Sarmin, Monira ; International Centre for Diarrhoeal Disease Research Bangladesh, Nutrition and Clinical Services Division<br>Shaly, Nusrat ; International Centre for Diarrhoeal Disease Research Bangladesh, Nutrition and Clinical Services Division<br>Sultana, Tania; International Centre for Diarrhoeal Disease Research Bangladesh, Nutrition and Clinical Services Division<br>Tariqujjaman, Md. ; International Centre for Diarrhoeal Disease Research Bangladesh<br>Shikha, Shamima ; International Centre for Diarrhoeal Disease Research Bangladesh, Nutrition and Clinical Services Division<br>Mariam, Nafisa; International Centre for Diarrhoeal Disease Research Bangladesh, Nutrition and Clinical Services Division<br>Jeorge, Didarul ; International Centre for Diarrhoeal Disease Research Bangladesh, Nutrition and Clinical Services Division<br>Tabassum, Mosharrat; International Centre for Diarrhoeal Disease Research Bangladesh, Nutrition and Clinical Services Division<br>Nahar, Baitun; International Centre for Diarrhoeal Disease Research Bangladesh<br>Afroze, Farzana; International Centre for Diarrhoeal Disease Research Bangladesh<br>Shahrin, Lubaba; International Centre for Diarrhoeal Disease Research Bangladesh, NCSD<br>Hossain, Md. Iqbal ; International Centre for Diarrhoeal Disease Research Bangladesh, Nutrition and Clinical Services Division<br>Alam, Baharul; International Centre for Diarrhoeal Disease Research Bangladesh, Nutrition and Clinical Services Division<br>Faruque, ASG; International Centre for Diarrhoeal Disease Research, Nutrition and Clinical Services Division<br>Islam, M; International Centre for Diarrhoeal Disease Research Bangladesh, Nutrition and Clinical Services Division<br>Osmany, Mohammad ; Bangabandhu Sheikh Mujib Medical University, Department of Cardiology<br>Chowdhury, Meshkat A.; Bangabandhu Sheikh Mujib Medical University, Department of Cardiology<br>Manji, Karim; Muhibili University of Health and Allied Sciences, Department of Pediatrics and Child Health<br>Kissoon, Niranjan; University of British Columbia, Department of

	Pediatrics<br>Chisti, Md.; International Centre for Diarrhoeal Disease Research Bangladesh, Nutrition and Clinical Services Division<br>Ahmed, Tahmeed; International Centre for Diarrhoeal Disease Research Bangladesh, Nutrition and Clinical Services Division
Keywords:	Paediatric intensive & critical care < ANAESTHETICS, Infectious diseases & infestations < DERMATOLOGY, Paediatric gastroenterology < PAEDIATRICS, Respiratory infections < THORACIC MEDICINE

SCHOLARONE™
Manuscripts

Title: Efficacy of dopamine, adrenaline and blood transfusion for treatment of fluid refractory shock in children with severe acute malnutrition or severe underweight and cholera or other dehydrating diarrheas: protocol for a randomized controlled clinical trial

Short Title: Efficacy of dopamine, adrenaline and blood transfusion for treatment of fluid refractory shock in severe malnutrition

For peer review only

Authors:

Monira Sarmin[1], Nusrat Jahan Shaly[1], Tania Sultana[1], Md Tariqujjaman[1], Shamima Sharmin Shikha[1], Nafisa Mariam[1], Didarul Haque Jeorge[1], Mosharrat Tabassum[1], Baitun Nahar[1], Farzana Afroze[1], Lubaba Shahrin[1], Md. Iqbal Hossain[1], Baharul Alam[1], ASG Faruque[1], Md. Munirul Islam[1], Din-E-Mujahid Mohammad Faruqe Osmany[2], Chaudhury Meshkat Ahmed[2], Karim P Manji[3], Niranjan Kissoon[4], Md. Jobayer Chisti[1], Tahmeed Ahmed[1]

Affiliations:

[1]Nutrition and Clinical Services Division, icddr,b, Dhaka, Bangladesh

[2]Department of Cardiology, Bangabandhu Sheikh Mujib Medical University (BSMMU), Dhaka, Bangladesh

[3]Department of Paediatrics and Child Health, Muhimbili University of Health and Allied Sciences, Dar es Salaam, Tanzania

[4]Department of Paediatrics, University of British Colombia & British Colombia Children's Hospital, Canada

Corresponding author: Md. Jobayer Chisti (chisti@icddrb.org)

For peer review only - http://bmjopen.bmj.com/site/about/guidelines.xhtml

ABSTRACT

Introduction Diarrhea is one of the leading causes of under-five childhood mortality and accounts for 8% of 5.4 million global under-5 deaths. In severely malnourished children, diarrrhea progresses to shock, where the risk of mortality is even higher. At icddr,b Dhaka Hospital, the fatality rate is as high as 69% in children with severe malnutrition and fluid refractory septic shock. To date, no study has evaluated systematically the effects of inotrope or vasopressor or blood transfusion in children with dehydrating diarrhea (for example, in cholera) and severe acute malnutrition (SAM) or severe underweight who are in shock and unresponsive to WHO-recommended fluid therapy. To reduce the mortality of severely malnourished children presenting with diarrhea and fluid refractory shock, we aim to compare the efficacy of blood transfusion, dopamine, and adrenaline in fluid refractory shock in children who do not respond to WHO-recommended fluid resuscitation.

Methods and analysis In this randomized, three-arm, controlled, non-masked clinical trial in children 1-59 months old with SAM or severe underweight and fluid refractory shock, we will compare the efficacy of dopamine or adrenaline administration versus blood transfusion in children who failed to respond to WHO-recommended fluid resuscitation. The primary outcome variable is the case fatality rate. The effect of the intervention will be assessed by performing an intention-to-treat analysis. Recruitment and data collection began in July 2021 and are now ongoing. Results are expected by May 2023.

Ethics and dissemination This study has been approved by the icddr,b Institutional Review Board. We adhere to the 'Declaration of Helsinki' (2000), guidelines for Good Clinical Practice. Before enrolment, we are collecting signed informed consent from the parents or caregivers of the children. We will publish the results in a peer-reviewed journal and will arrange a dissemination seminar.

Trial registration number ClinicalTrials.gov**;** NCT04750070, February 11, 2021

Version no:2.2, Dated July 21, 2022

For peer review only

STRENGTHS AND LIMITATIONS OF THIS STUDY

⇒ This trial is expected to provide valuable data to determine the best possible resuscitation strategies- dopamine or adrenaline or blood in the management of fluid refractory shock in SAM or severely underweight children.

⇒This study includes malnourished children having fluid refractory shock and diarrhea

⇒This study evaluates cardiac function of the study children at bedside by point-of-care (POC) cardiac USG

⇒ This study is excluding children having congenital heart diseases, Down's syndrome or any chronic illness

⇒ Evaluation of non-diarrheal children having shock is out of scope for this study

INTRODUCTION

Background and rationale

Diarrheal diseases are the second leading cause of death in under-five children and were responsible for the deaths of 370,000 children in 2019 [1]. In Bangladesh, diarrhea causes 7.3% of under-5 deaths in 2019 [2]. Children with severe acute malnutrition (SAM) frequently present with diarrhea, and fluid refractory shock is one of the common complications of diarrhea [3]. The management of these children is challenging. There is a lack of evidence-based guidelines for the management of fluid refractory shock in severely malnourished children. Optimized management of the altered hemodynamics in fluid refractory shock is yet to be explored and different opinions exist among experts, especially for malnourished children. Data from the Dhaka Hospital of icddr,b demonstrates that despite appropriate care, 69% of children having co-morbidities such as severe malnutrition die from fluid refractory shock [4].

The World Health Organization (WHO) recommends administration of isotonic saline boluses in a dose of 15 mL/kg over 1 hour (maximum two times), followed by blood transfusion in a dose of 10 mL/kg over 3 hours in cases unresponsive to isotonic fluid boluses and have a fluid refractory/septic shock [5]. Quick arrangement of safe blood is difficult, especially in resource-poor settings. The FEAST trial showed significantly higher mortality associated with the administration of saline bolus or albumin [6] in children having severe sepsis. Inotropes like dopamine perform better than excessive fluid administration [7] when myocardial dysfunction or vasoregulatory failure results in shock rather than absolute hypovolemia [7 8]. Recent studies also demonstrated that adrenaline resulted in the early resolution of shock with survival benefits than dopamine [9 10]. Despite the very high death rates, the management of fluid refractory shock in severely malnourished children with early inotropes or vasopressors has never been studied.

For peer review only - http://bmjopen.bmj.com/site/about/guidelines.xhtml

Malnourished children have less left ventricular mass [11 12] as well as left ventricular function as evidenced by echocardiography [11 13] which may constitute a bad prognostic parameter. The FEAST trial reported that in the bolus group a significantly higher proportion had a terminal event cardiac rather than respiratory or neurological [14]. Point of care (POC) cardiac ultrasound is an emerging tool to assess cardiac function and fluid responsiveness in shock and it is non-invasive. The use of the POC ultrasound to differentiate hypovolemic shock from distributive shock e.g. fluid refractory/septic shock, by measuring ventricular function and IVC collapsibility can reduce the diagnostic time as well as increase the accuracy of diagnosis [15 16]. Usually, in hypovolemic shock, both end-diastolic and end-systolic volume are reduced, [16] left ventricle (LV) is small having a hyperdynamic wall motion [17]. Whereas in fluid refractory/septic shock, LV is hyperdynamic[17] and only end-systolic volume is reduced [16]. Again, the diameter of the IVC at inspiration and expiration and their ratio provides useful information regarding fluid responsiveness [16]. To date, no study has evaluated systematically the effects of vasopressor and inotrope(s) in children with dehydrating diarrhea (for example, in cholera) and SAM having fluid refractory shock unresponsive to conventional WHO-recommended fluid therapy.

The proposed study will provide valuable data to determine whether resuscitation strategies including vasopressor and inotropes or blood results in increased survival in children who are severely malnourished having fluid refractory shock and diarrhea. In addition, POC cardiac ultrasound will provide information on whether the shock resulted from a fluid deficit or cardiac dysfunction.

Objective

The study aims to compare the efficacy of blood transfusion, dopamine, and adrenaline in fluid refractory shock in children having SAM or severe underweight who do not respond to WHO-recommended fluid resuscitation.

Trial design

This is a randomized, three-arm, controlled, non-masked clinical trial in children 1-59 months old with SAM or severely underweight and shock (Figure 1). With parental written informed consent, children are allocated to the study interventions following randomization, in addition to usual supportive care that includes broad-spectrum antibiotics, oxygen inhalation, IV glucose, etc. The study protocol follows the Standard Protocol Items: Recommendations for Interventional Trials (SPIRIT) guidance for protocol reporting (modified to reflect the ongoing study activities) [18] (Table 1 and Figure 2).

For peer review only - http://bmjopen.bmj.com/site/about/guidelines.xhtml

METHODS

Participants and interventions and outcomes

Study setting

The study is being conducted at the Dhaka Hospital of the icddr,b, Dhaka, Bangladesh. icddr,b is the largest diarrheal diseases hospital in the world. Annually a total of 194,838 patients of all ages and both genders got treatment and care from icddr,b, and 57.2% of them were under five years of age. Besides diarrheal illness, many patients have other health conditions, like pneumonia, malnutrition, sepsis, and electrolyte abnormalities. For critically ill patients, there is an intensive care unit facility present within the hospital, equipped with contemporary life support measures, including mechanical ventilators, bubble continuous positive airway pressure (BCPAP) oxygen therapy, high flow nasal cannula (HNFC) oxygen therapy, and syringe pumps for vasopressor support. The ICU of the Dhaka Hospital of icddr,b manages around 1000 pediatric patients each year. More than 80% of them have a poor socio-economic background. About one-third have severe malnutrition, and one-fourth of them have severe sepsis or fluid refractory shock, there are other different wards for the treatment of diarrheal illness, respiratory problems, and malnutrition. icddr,b possesses a well-equipped laboratory service capable of performing a range of clinical tests proposed in this study.

Inclusion criteria are children aged 1-59 months of either sex having diarrhea and SAM or severe underweight who develop fluid refractory shock either at admission or at any point during their treatment in the hospital (which might be due to cholera or other dehydrating diarrhea or severe sepsis)

Exclusion criteria are severe anemia (hemoglobin < 5 gm/dl) and sclerema where blood transfusion is a mandate, congenital anomalies (tetralogy of Fallot (TOF)/atrial or ventricular septal defect (ASD or VSD)/Trisomy 21, etc.), negative blood groups, a requirement of cardio-pulmonary resuscitation during screening and non-consent from the caregivers/parents.

Randomization: The permuted block randomization technique is followed to select treatment arms for each child. The randomization procedure was planned and executed by a scientist from icddr,b who is not involved in the study. The randomization list containing the subject identification numbers (IDs) and the corresponding group allocation remained concealed. IDs are chronologically assigned to each new study participant. After randomization, an opaque envelope containing the name of the allocated intervention group is opened.

For peer review only - http://bmjopen.bmj.com/site/about/guidelines.xhtml

Table 1: Standard Protocol Items: Recommendation for Interventional Trials (SPIRIT) Figure. The schedule of enrolment, interventions, and assessments

**Indicators**		**Study period**						
	Screening	Enrolment & allocation	Study drugs administration & F-U	Rescue therapy	Hosp course up to disposal	Follow up after discharge		
						1st follow up	2nd follow up	28th day follow up (over phone)
Time point	Day 0	Hour 0	Hour 0-3 (1 hour for Dopamine & Adrenaline, 3 hours for Blood)	Hour 1- disposal (for Dopamine & Adrenaline) Hour 3- disposal (for Blood)		Day 7±2	Day 21±2	Day 28±3
Enrolment								
Eligibility screen	x							
Informed consent	x	x						
Randomization and allocation		x	x					
Interventions								
Different drugs (Dopamine or Adrenaline or Blood)			x					
Assessments:								
Demographic		x						
Comorbidities		x						
Physical examination	x	x	x	x	x	x	x	-
Primary outcome:								
case fatality								x
Secondary outcomes:								
Treatment failure			x					
MAP stabilization at 48 hours after enrolment				x	x			

For peer review only - http://bmjopen.bmj.com/site/about/guidelines.xhtml

Mechanical ventilation			x	x	x			
Incidence of heart failure			x	x	x			
Duration of ICU stay					x			
Duration of total hospitalization					x			
Time-to-achieve recovery from shock			x	x	x			
Ventricular function by cardiac USG	x	x		x	x (if require)			
**IVC (Inferior Vena Cava) collapsibility**	x	x		x	x (if require)			
[a]Detection of leptin from stored blood		x						

[a]According to the addendum (ERC approval on October 21 2021), at the end of the study, stored blood samples will be tested for leptin.

Collection of Baseline Information

Any SAM children with fluid refractory shock, either at admission or developed at some point during their treatment period, within the defined age group are screened for study eligibility criteria by the study physician. According to the inclusion and exclusion criteria, parents or attending caregivers of those eligible children are invited for providing consent for their children to be enrolled in the study. A signed written informed consent form by parents or caregivers gives data concerning the study, attainable advantages and risks, and voluntary nature of participation, as well as the right to cancel the child's participation in the study at any time (even after giving the initial consent) while not providing any reason; after this, children are enrolled by the study physician. One copy of the signed consent document was given to the caregiver of every participant and another copy was reserved for the study documentation. A pretested case record form is used to collect relevant medical history information, including the nature and duration of illness, medication for current illness, and any history of significant past illness including pneumonia and diarrhea. The form is also used to collect information on sociodemographic characteristics, such as age, sex, religion, gestational age, parental age with education and occupations, source of drinking water and sanitation, type of fuel use, cooking place and smoking history, average monthly income of the family, number of siblings, and number of rooms in the home. Information is also collected about each child's feeding practice, which included their history of breastfeeding, formula feedings, or alternative complementary feedings, and also immunization status.

For peer review only - http://bmjopen.bmj.com/site/about/guidelines.xhtml

Data on clinical examination findings are recorded by the study physician and include pulse, respiratory rate, axillary temperature, anthropometric measurements (ie, height, weight, mid-upper arm circumference for calculation of Z score), chest auscultation, oxygen saturation, presence or absence of chest wall indrawing, cyanosis, and mental status (ie, normal, irritable, or lethargic), and response according to AVPU. Each child's weight is measured by using an electronic weighing scale with a precision of 0.1 kg; length is measured using a standard-length board with a precision of 0.5 cm and mid-upper arm- circumference (MUAC) is measured using a standard MUAC tape with a precision of 0.2 cm by the trained and experienced study nurse of Dhaka hospital. Fever is defined when the axillary temperature is 38°C or greater. Respiratory rate is counted for a full 60 seconds by exposing the trunk when the child is awake and calm; the presence of lower-chest-wall indrawing is noted at the same time. Oxygen saturation is measured using a portable pulse oximeter (Handheld Vital Signs Monitors- NT1D, Solaris Medical Technology, Inc, USA) with a probe on a finger or toe when the child breathes in room air. Oxygen saturation of <90% is defined as hypoxemia, which is an indication for supplemental oxygen therapy by using BCPAP. Blood group and Rh typing is done at the bedside and we exclude the child having negative blood groups.

Bedside Hb (if not known) is checked by using Hemocue analyser during the screening period to exclude severely anemic children requiring an urgent blood transfusion. The length of ICU stays, the total length of hospitalization and time-to-achieve-recovery (time required for the restoration of MAP after the starting of the intervention for fluid refractory shock) are recorded. We are also keeping a record of stool and urine volume. Any deterioration or adverse events (ie, signs of heart failure or need for mechanical ventilation) are also documented. Both the study and outcome are recorded including treatment failure/success, hospital discharge, referrals, and deaths or left against medical advice (LAMA).

Laboratory Testing and sample handling

We are performing laboratory tests after the enrolment of a child in the study that includes blood for haemoglobin percentage, total and differential counts of white blood cell (WBC), hematocrit, blood culture and antimicrobial susceptibility of pathogens, serum electrolytes and creatinine, calcium and magnesium, serum bilirubin, serum lactate, C-reactive protein (CRP), procalcitonin, and cardiac troponin I, stool (rectal swab for Cholera, Salmonella, Shigella and Campylobacter) and urine culture, chest radiograph, and arterial or capillary blood gas analysis. We are also doing a test for the exclusion of dengue during the dengue prevalent period in the country. We are checking serum lactate at enrolment and 4-6 hours after enrolment. Our target is to send samples for laboratory tests within 30 min-1 hour of enrolment. When a child is in shock, very often it is difficult to get an adequate amount of blood for

For peer review only - http://bmjopen.bmj.com/site/about/guidelines.xhtml

proposed testing. Then we prioritize tests based on clinical relevance. We are doing the blood grouping, screening and cross-matching while we are following a child for study eligibility. We are performing cardiac ultrasonography during screening, enrolment and 4-6 hours later after enrolment. We are archiving blood samples and planning to check serum leptin at the end of the study.

Study intervention and procedure

This is a three-arm trial where the intervention arms (dopamine and adrenaline) will be compared with the WHO standard intervention (blood transfusion). Children with fluid refractory shock are randomized to blood transfusion, dopamine, or adrenaline therapy. During the screening, children receive intravenous isotonic fluid @ of 15mL/kg over one hour (maximum two boluses i.e. up to 30ml/kg) as per WHO standard of care. The choice of isotonic fluid is Ringer`s lactate/Hartsol (Normal saline if any suspicion of hyperkelemia, acetate if severe dehydration is predominant). Children in the dopamine arm (**Treatment category A**) receive dopamine, 8 microgram/kg.min (increasing the dose after 15 minutes to 12 microgram/kg/min to a maximum of 15 microgram/kg/min, up to the achievement of goal). Children in the adrenaline arm (**Treatment category B**) receive adrenaline, 0.1 microgram/kg/min (increasing the dose after 15 minutes to 0.2 microgram/kg.min to a maximum of 0.3 microgram/kg.min, up to the achievement of goal). Children in the blood transfusion arm (**Treatment category C)** receive a transfusion of whole human blood in a dose of 10 mL/kg over 3 hours (Figure 1). If blood could not be arranged within one hour after the event of fluid refractory shock, we will start rescue therapy. In addition to the study interventions, broad-spectrum antibiotic therapy, oxygen therapy, and frequent monitoring, all children receive IV maintenance fluids @ of 3 ml per kg per hour during the arrangement of blood transfusion and intervention with inotropes. IV fluid maintenance is continued for 6 hours after the achievement of a goal and is further continued if the goal is not achieved. Our goal is to achieve MAP >50 mm of Hg and/or urine output ≥1 ml/kg per hour. After randomization, we monitor children at every 15-minute interval during the intervention to assess mental status [according to AVPU, an acronym from "alert, verbal, pain, unresponsive"], capillary refill time, rate and volume of the radial pulse, cold/warm extremities, urine output, blood pressure, respiratory rate, peripheral capillary O_2 saturation, and features of heart failure. Mechanical ventilation is provided if the child did not respond to bubble CPAP (if SpO2<85% at least after an hour of bubble CPAP oxygen therapy) or had a respiratory failure (Ph<7.2 and PCO_2>60 mm of Hg in ABG analysis or SpO_2/FiO_2<265). Children who develop complications such as acute abdomen not responding to conservative management or acute renal insufficiency requiring dialysis are referred to a suitable higher facility for proper management. At the achievement of the goal, we continue the dose of dopamine or adrenaline for 6 hours. We follow up with the child every 30 min to

For peer review only - http://bmjopen.bmj.com/site/about/guidelines.xhtml

1-hour interval. Thereafter we reduce the dose in a stepwise manner similar to how we increased the dose. If the child becomes hypotensive again, we re-introduce the previous dose and follow the same procedure for further iteration.

Rescue therapy

The failure of any intervention is declared as a treatment failure. Then for children in dopamine and blood transfusion arm, we start adrenaline as rescue therapy in a stepwise manner starting from 0.1 microgram/kg/min and increasing every 15 min by 0.1 microgram/kg/min up to restoration of the desired goal (MAP >50 mm of Hg or urine output>1ml/kg/hour) and the highest dose is 0.5 microgram/kg/min (or consultant discretion) For children in the adrenaline arm, the dose is increased every 15 minutes from 0.3 microgram/kg/min (the highest proposed intervention dose) to a max of 0.5 microgram/kg/min. Upon achieving the goal, we continue the same dose for 4-6 hours and then we de-escalate in a stepwise manner.

If the goal is not achieved with injection of adrenaline then injection of noradrenaline is started @ 0.1 microgram/kg/min and we increase the dose every 15 min by 0.1 microgram/kg up to restoration of the desired goal (MAP >50 mm of Hg or urine output>1ml/kg/hour) and the highest dose is 0.5 microgram/kg/min. Upon achieving the goal, we continue the same dose for 6 hours and then will de-escalate in a stepwise manner. For those patients whose MAP is not restored with inotropes, injection hydrocortisone @ 2mg/kg every 6 hours is added [19]. During rescue therapy, for doses and selection of any inotrope(s), we are following the recommendation from the PICU consultant as all cases are not the same.

Feeding and other care

Depending on the clinical condition either we keep the child NPO or start trophic feeding for children who had MAP >50 mm of Hg and had no evidence of paralytic ileus. Feeds consist of "Milk Suzi" (similar to WHO recommended F-75) for children older than 6 months, and modified infant formula (in addition to breast milk, if applicable) is given to infants 1-6 months of age [20]. Thus, we provide a fluid volume of 120ml/kg/day to deliver 80-82 Kcal/kg/day. A child is kept warm to prevent hypothermia. If there is hypoglycaemia (random blood glucose < 3mmol/L), correction is given with 10% glucose intravenously in a dose of 5 mL/kg to be followed by 10% glucose administration through a nasogastric tube, following the standard protocol of the hospital.

For peer review only - http://bmjopen.bmj.com/site/about/guidelines.xhtml

Nutritional rehabilitation

As the study children have severe acute malnutrition, they require nutritional rehabilitation. After recovering from acute illness, the mothers/caregivers are asked to stay in the hospital Nutritional Rehabilitation Unit (NRU) for 2-3 weeks. In the NRU, the standard dietary protocol is followed for nutritional rehabilitation and the children are discharged upon fulfilling discharge criteria.

Post-discharge follow-up

We follow up with a child on the 28th day (±3 days) of enrolment to know their clinical status over the phone. In addition, the children who were discharged from NRU or taking early discharge from the ward, are requested to come for a pre-scheduled follow-up at the hospital nutrition follow-up unit (NFU) of icddr,b. We followed the children at NFU and record their nutritional status and health problems. Our scheduled follow-ups for this study children are in two-time points; once after 7 days of discharge, then after 15 days of 1st follow-up. If any mother/caregiver fails to attend a follow-up visit, then we communicate with the mother/caregiver over the phone for two-time points. We document the outcome whether they are alive or not, and had any intercurrent illness e.g. diarrhea, pneumonia.

Outcome measures

Primary outcome variable is the case fatality rate (at 28±3 days from the day of enrolment). The secondary outcome variables are the rate of treatment failure, rate of MAP stabilization at 48 hours after enrolment, need for mechanical ventilation, the incidence of heart failure, duration of ICU stay and total hospitalization, time-to-achieve recovery, evaluation of cardiac function by point of care ultrasonogram and Inferior Vena Cava (IVC) collapsibility.

Sample size

The 2010-11 data from the ICU of the Dhaka Hospital of the icddr,b (the proposed study site) revealed that among the 36 severely malnourished children with shock, 22 died i.e. mortality rate among SAM children with septic shock was 61% (22/36). We assumed that the proposed interventions would reduce the case fatality rate by 50%, i.e. the rate will decrease from 61% to 31%.
To detect this magnitude of difference in the death rate, with 80% power (20% type II error) and 0.05 type I error, the required sample size is 40 children in each of the three groups. Considering around 10%

For peer review only - http://bmjopen.bmj.com/site/about/guidelines.xhtml

dropouts after enrolment, the total sample size would be 45 children at least in each of the three groups, i.e. a total of 135 children - the final sample size of our study.

Data collection, management and analysis

Data will be collected using a hard copy structured questionnaire. Data will be entered into a personal computer on the online platform, REDCap. The data management team will download and store the dataset as a backup to ensure data security. Statistical analyses will include descriptive as well as analytical methods. The effect of the intervention will be assessed by performing an intention-to-treat analysis as well as a per-protocol analysis; we anticipate that there will be no differences in the baseline data among the groups. Data analysis will be done using SPSS for Windows (Version 20.0. Armonk, NY: IBM Corp and Epi Info (version 7.0, USD, Stone Mountain, GA) or STATA. Categorical variables will be compared using the chi-square test or Fisher exact test as appropriate. When the variables of interest are continuous and parametric, the statistical significance of group means comparisons will be evaluated by ANOVA. When the main outcome measures are continuous nonparametric variables, the statistical significance of differences will be determined by the Kruskal-Wallis test. The post hoc test will be done if required. A probability of less than 0.05 will be considered statistically significant. The strength of association will be determined by calculating the relative risk (RR) and their 95% confidence intervals (CI). Finally, regression analyses will be performed to reach more definitive conclusions. In addition, we will analyze the Kaplan-Meier survival curve to compare the survival times among the three treatment groups. If any difference in baseline data, regression analyses will be performed for more definitive conclusions after adjusting for potential confounders. The schedule interim analysis will be done after 50% of enrolment or earlier if DSMB advise. We will follow the stopping rule if the $P < 0.003$, as recommended by Pato and Fleming [21].

Reporting of adverse events

Serious adverse events (deaths) after initiation of the study intervention are recorded and presented to the DSMB within 24 hours of the event. The report includes a detailed clinical history, the study intervention, and management given until the outcome. We contacted over the phone for study participants who are referred.

For peer review only - http://bmjopen.bmj.com/site/about/guidelines.xhtml

Operational definition

Severe Acute Malnutrition (SAM): A child with a <-3 Z-score of weight for height/length (WHZ/WLZ) of the median value of the WHO standard or MUAC<115 mm (for children >6 months of age) /visible wasting (for children <6 months of age), or presence of nutritional edema.

Severe underweight: A child with a <-3 Z-score of weight for age (WAZ) of the median value of the WHO standard.

Sepsis: Presence of infection/inflammation plus hyperthermia or hypothermia (temperature >38.5^{o} Celsius or <35.0^{o} Celsius respectively) plus tachycardia (HR: infant>160/min, 1-5 years>140/min) or abnormal WBC count ($>12\times10^{9}/L$* or, $<4\times10^{9}/L$ or, band and neutrophil ratio ≥ 0.1)

Severe sepsis: Severe sepsis is defined as the presence of sepsis plus organ dysfunction or tissue hypoperfusion [poor peripheral perfusion (hypotension and/ or absent peripheral pulses and/or delayed capillary refilling time in the absence of dehydration or after correction of dehydration)]

Septic Shock: Shock is defined hypotension (Mean arterial pressure <50mm of Hg) and or reduced urine output <1 mL/kg/h despite fluid resuscitation [22].

Mean arterial pressure (MAP): MAP is calculated from systolic blood pressure (SBP) and diastolic blood pressure (DBP) by using a formula: DBP added to 1/3rd of pulse pressure (subtraction of the DBP from the SBP).

Treatment failure: Treatment failure is defined if an intervention results in

- ☐ MAP < 50 mm of Hg or
- ☐ Urine output is < 1 ml/kg per hour, at the end of intended intervention over 2/3 hour

Heart failure (a combination of findings)

- ☐ Age-specific tachypnea
- ☐ Tachycardia
- ☐ Enlarged tender liver
- ☐ Pedal edema (new or worsening)
- ☐ Basal crackles and/or
- ☐ Gallop and
- ☐ Response to furosemide

Signs of improvement (during 1st hour/2nd hour of fluid bolus therapy)

- ☐ Pulse rate falls
- ☐ Pulse volume improves
- ☐ Respiratory rate falls

For peer review only - http://bmjopen.bmj.com/site/about/guidelines.xhtml

For peer review only

- ☐ No signs of pulmonary edema

Signs of deterioration (during 1st hour/2nd hour of fluid bolus therapy)

- ☐ Breathing rate increases by 5/minute
- ☐ Pulse rate increases by 15/minute
- ☐ Liver enlarges (by measuring size at the beginning of fluid resuscitation and at the time of deterioration)
- ☐ Fine crackles at the lung base or throughout the lung fields
- ☐ Edema (new or worsening)
- ☐ Galloping heart rhythm

Acute kidney injury (AKI): AKI is defined as an increase in serum creatinine by ≥ 0.3 mg/dL from baseline within 48 hours OR, an increase in serum creatinine to ≥ 1.5 times baseline or age-specific upper limit of reference interval (ULRI) OR, urine volume ≤ 0.5 mL/kg/hour for six hours.

Recovery time: Time required for the restoration of MAP after starting the intervention for shock

Congenital heart diseases: Atrial septal defect, ventricular septal defect, Tetralogy of Fallot (TOF)

Organic/other diseases: Down`s syndrome, cerebral palsy

Patient and public involvement

We have planned to conduct public engagement activities like round table discussions and workshops at the end of the study.

Ethics

This study has been approved by the Institutional Review Board (IRB) of the icddr,b (Protocol No. PR#20021, version 2.0, dated 06.10.2021). The study is performing in compliance with the 'Declaration of Helsinki' (2000), Guideline for Good Clinical Practice (GCP). Before enrolment, we are collecting signed informed consent from the parents or caregivers of the children. We are strictly maintaining privacy, anonymity, and confidentiality of data or information identifying the patients or caregivers. Participants' medical information, particulars of treatment, and laboratory test results will be kept confidential and under lock and key; only the research staff will have access to this information. The risk to enrolled subjects from participation in the study is minimal. Adverse medical events are anticipated as the study is enrolling critically ill patients. This protocol has been reviewed & approved by the Data Safety and Monitoring Board (DSMB) of icddr,b. DSMB is a five membered board who independently review the activity of study. We are reporting the occurrence of any serious adverse event (e.g., death) to the DSMB within 24 hours of the event and will follow their recommendations. There is no influence of

For peer review only - http://bmjopen.bmj.com/site/about/guidelines.xhtml

the funder at any stage of the research, starting from study design and up to publication. Study patients are recruited into three different groups. For this clinical trial, inj adrenaline and inj dopamine are being used in fluid refractory shock. These drugs are already in use in the ICU of Dhaka hospital of icddr,b [22]. WHO recommends blood transfusion, and we are routinely practicing it. Regardless of the study result, finally, it will be disseminated.

Strategies for achieving adequate participant enrolment

Treatment at icddr,b Dhaka Hospital is free of cost for all patients. In addition to routine care, the study children get more follow up by study physicians. So, they get the benefit of early detection and early intervention for any clinical condition. The caregiver has access to the study investigators and other physicians to communicate and to get an answer to their questions related to their child's medical condition and treatment even after their discharge. However, we pay a small amount of money (transport cost) for their follow-up visit after their usual discharge. We believe this information and empathetic care from the study will ensure adequate participant enrolment to reach the target sample size and also reduce the number of lost to follow up.

Dissemination

After approval from the investigators of the protocol, we will publish the results and other findings of the study. We will use The International Committee of Medical Journal Editors guidelines to ascertain the authorship of papers.

Study status: As of August 2022, we have screened 49 children and enrolled 18 participants. Participants screening and enrolment are ongoing.

Protocol amendments

After the first approval of the protocol, we included two cardiologists as co-investigators. After one year of enrolment we made change in the inclusion criteria also. Now we are including severe underweight children along with SAM children. So, we required amendments of the protocol which were all reviewed and approved by the icddr,b IRB. Now we are using version 2.2 of the protocol.

DISCUSSION

The purpose of this clinical research is to find evidence-based management for the treatment of fluid refractory shock among children having SAM or severe underweight and diarrhea.

For peer review only - http://bmjopen.bmj.com/site/about/guidelines.xhtml

The data gathered from this study will hopefully be of great interest to scientists either to modify the management strategy of fluid refractory shock or to pursue new elements in the management of fluid refractory shock. Here we will use POC USG to evaluate the cardiac function and volume status of a child having fluid refractory shock.

In conclusion, this study is expected to provide useful insights into the efficacy of dopamine, adrenaline and blood transfusion for the treatment of fluid refractory shock in children with severe acute malnutrition and cholera or other dehydrating diarrheas. If any intervention works better in this setting, we could plan to incorporate this in the management plan for our patients visiting ICU, icddr,b Dhaka hospital to observe the real-world scenario beyond the boundary of a controlled clinical trial. We would have scope to examine this study in other countries where SAM/severe underweight is prevalent.

Contributorship statement

The study concept and design were conceived by MS, BN, LS, MIH, BA, ASGF, MMI, DEMMFO, CMA, KPM, NK, MJC and TA. NJS, TA, SSS, NM, DHJ, MT, and MS are conducting patient's screening, patient's management and data collection. Data management is performed by MT. Analysis will be performed by MT, MS, MJC, TA. MS prepared the first draft of the manuscript. All authors reviewed, edited and critiqued the manuscript.

Competing interest

None declared

Funding

This protocol is supported by Wellcome Trust, Grant number- 215691/Z/19/Z.

Acknowledgement

icddr,b is grateful to the governments of Bangladesh, Canada, Sweden and the UK for providing core/unrestricted support.

References

1. WHO. Diarrhoea. 2022
2. Unicef. Diarrhoea 2022 [Available from: https://data.unicef.org/topic/child-health/diarrhoeal-disease accessed 26 April 2022].

For peer review only - http://bmjopen.bmj.com/site/about/guidelines.xhtml

3. Chisti MJ, Pietroni MA, Smith JH, et al. Predictors of death in under-five children with diarrhoea admitted to a critical care ward in an urban hospital in Bangladesh. *Acta paediatrica* 2011;100(12):e275-e79.
4. Chisti MJ, Salam MA, Bardhan PK, et al. Severe sepsis in severely malnourished young Bangladeshi children with pneumonia: A retrospective case control study. *PloS one* 2015;10(10):e0139966.
5. Organization WH. Pocket book of hospital care for children: guidelines for the management of common childhood illnesses: World Health Organization 2013.
6. Maitland K, Kiguli S, Opoka RO, et al. Mortality after fluid bolus in African children with severe infection. *New England Journal of Medicine* 2011;364(26):2483-95.
7. Brierley J, Carcillo JA, Choong K, et al. Clinical practice parameters for hemodynamic support of pediatric and neonatal septic shock: 2007 update from the American College of Critical Care Medicine. *Critical care medicine* 2009;37(2):666.
8. Gill A, Weindling A. Randomised controlled trial of plasma protein fraction versus dopamine in hypotensive very low birthweight infants. *Archives of disease in childhood* 1993;69(3 Spec No):284-87.
9. Ventura AM, Shieh HH, Bousso A, et al. Double-Blind Prospective Randomized Controlled Trial of Dopamine Versus Epinephrine as First-Line Vasoactive Drugs in Pediatric Septic Shock. *Critical care medicine* 2015;43(11):2292-302. doi: 10.1097/ccm.0000000000001260 [published Online First: 2015/09/01]
10. Ramaswamy KN, Singhi S, Jayashree M, et al. Double-Blind Randomized Clinical Trial Comparing Dopamine and Epinephrine in Pediatric Fluid-Refractory Hypotensive Septic Shock. *Pediatr Crit Care Med* 2016;17(11):e502-e12. doi: 10.1097/pcc.0000000000000954 [published Online First: 2016/09/28]
11. ABUFADDAN N, Khalid E, Shams H, et al. Myocardial dysfunction in malnourished children. *Journal of Pediatric Sciences* 2010;2(2)
12. Olivares JL, Vazquez M, Rodríguez G, et al. Electrocardiographic and echocardiographic findings in malnourished children. *Journal of the American College of Nutrition* 2005;24(1):38-43.
13. El-Sayed H, Nassar M, Habib N, et al. Structural and functional affection of the heart in protein energy malnutrition patients on admission and after nutritional recovery. *European journal of clinical nutrition* 2006;60(4):502.
14. Maitland K, George EC, Evans JA, et al. Exploring mechanisms of excess mortality with early fluid resuscitation: insights from the FEAST trial. *BMC Med* 2013;11:68. doi: 10.1186/1741-7015-11-68 [published Online First: 2013/03/19]
15. Jones AE, Tayal VS, Sullivan DM, et al. Randomized, controlled trial of immediate versus delayed goal-directed ultrasound to identify the cause of nontraumatic hypotension in emergency department patients. *Critical care medicine* 2004;32(8):1703-8. doi: 10.1097/01.ccm.0000133017.34137.82 [published Online First: 2004/08/03]
16. Sun J-T. New Advances in Emergency Ultrasound Protocols for Shock. *J Med Ultrasound* 2017;25(4):191-94. doi: 10.1016/j.jmu.2017.09.005 [published Online First: 12/06]
17. Atkinson PRT, McAuley DJ, Kendall RJ, et al. Abdominal and Cardiac Evaluation with Sonography in Shock (ACES): an approach by emergency physicians for the use of ultrasound in patients with undifferentiated hypotension. *Emergency Medicine Journal* 2009;26(2):87-91. doi: 10.1136/emj.2007.056242

For peer review only - http://bmjopen.bmj.com/site/about/guidelines.xhtml

18. Chan A, Tetzlaff J, Altman D, et al. Krle a-Jeri K, et al. SPIRIT 2013 statement: defining standard protocol items for clinical trials. *Annals of Internal Medicine* 2013;158(3):200-7.
19. Weiss SL, Peters MJ, Alhazzani W, et al. Surviving sepsis campaign international guidelines for the management of septic shock and sepsis-associated organ dysfunction in children. *Intensive care medicine* 2020;46(1):10-67.
20. Ahmed T, Ali M, Ullah MM, et al. Mortality in severely malnourished children with diarrhoea and use of a standardised management protocol. *The Lancet* 1999;353(9168):1919-22.
21. Science PWCo. Frequentist Methods: O'Brien-Fleming, Pocock, Haybittle-Peto [Available from: https://online.stat.psu.edu/stat509/node/80/.

22. Sarmin M, Ahmed T, Bardhan PK, et al. Specialist hospital study shows that septic shock and drowsiness predict mortality in children under five with diarrhoea. *Acta Paediatr* 2014;103(7):e306-11. doi: 10.1111/apa.12640 [published Online First: 2014/03/26]

For peer review only

For peer review only - http://bmjopen.bmj.com/site/about/guidelines.xhtml

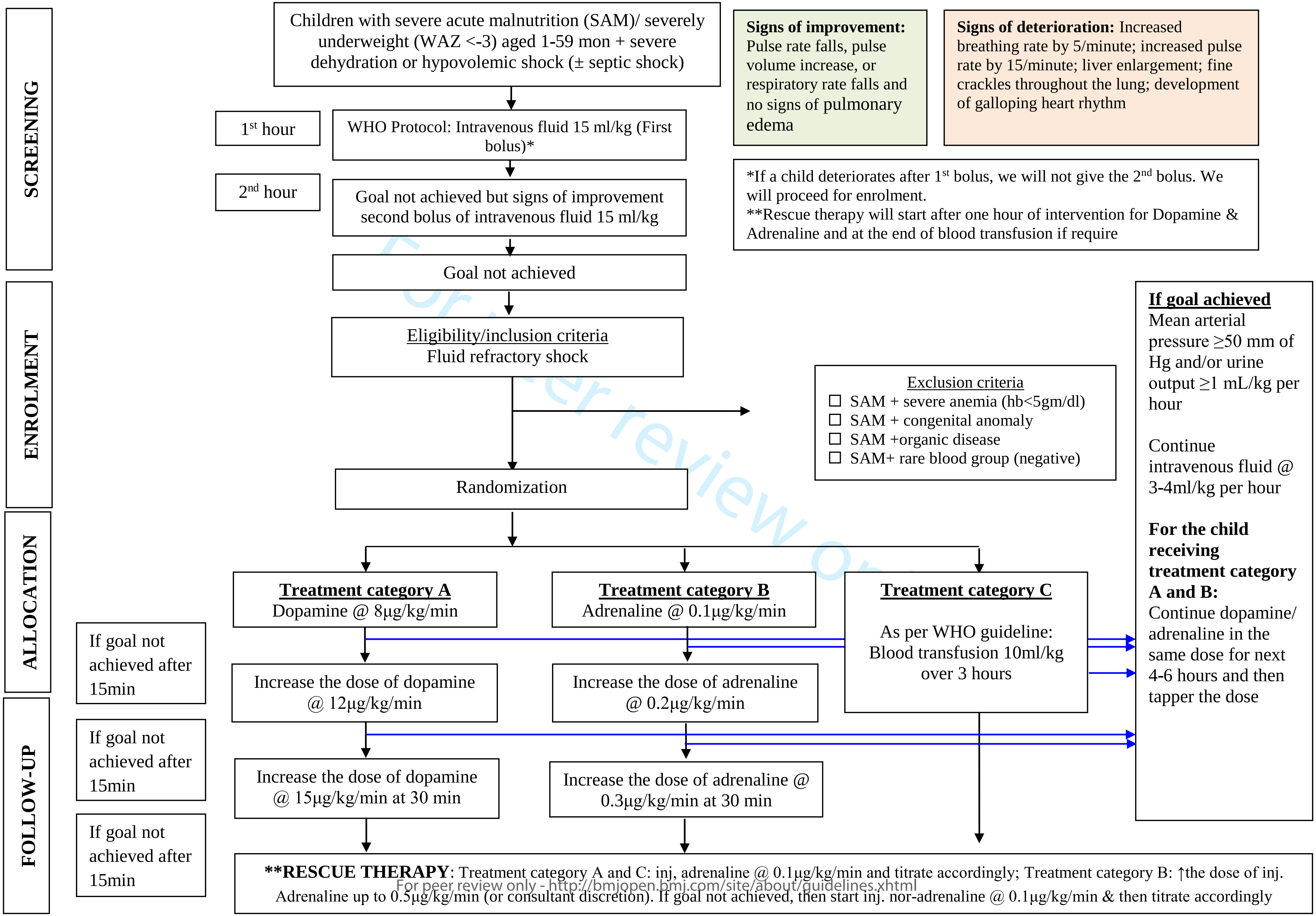

For peer review only - http://bmjopen.bmj.com/site/about/guidelines.xhtml

Figure 2: SPIRIT 2013 Checklist: Recommended items addressed in the clinical trial "Management of fluid refractory shock in children with SAM or severe underweight and diarrhea" protocol and related documents

Section/ item	Item No	Description	Addressed on page number
**Administrative information**			
Title	1	Descriptive title identifying the study design, population, interventions, and, if applicable, trial acronym	1
Trial registration	2a	Trial identifier and registry name. If not yet registered, name of intended registry	ClinicalTrials.gov, ID: NCT (page 2)
	2b	All items from the World Health Organization Trial Registration Data Set	-
Protocol version	3	Date and version identifier	2
Funding	4	Sources and types of financial, material, and other support	16
Roles and responsibilities	5a	Names, affiliations, and roles of protocol contributors	1
	5b	Name and contact information for the trial sponsor	icddr,b
	5c	Role of study sponsor and funders, if any, in study design; collection, management, analysis, and interpretation of data; writing of the report; and the decision to submit the report for publication, including whether they will have ultimate authority over any of these activities	3
	5d	Composition, roles, and responsibilities of the coordinating centre, steering committee, endpoint adjudication committee, data management team, and other individuals or groups overseeing the trial, if applicable (see Item 21a for data monitoring committee)	IRB, icddr,b (Page 14)

For peer review only

**Introduction**			
Background and rationale	6a	Description of research question and justification for undertaking the trial, including summary of relevant studies (published and unpublished) examining benefits and harms for each intervention	2,3,4
	6b	Explanation for choice of comparators	2,3,4
Objectives	7	Specific objectives or hypotheses	4,
Trial design	8	Description of trial design including type of trial (eg, parallel group, crossover, factorial, single group), allocation ratio, and framework (eg, superiority, equivalence, non-inferiority, exploratory)	4
**Methods: Participants, interventions, and outcomes**			
Study setting	9	Description of study settings (eg, community clinic, academic hospital) and list of countries where data will be collected. Reference to where list of study sites can be obtained	5
Eligibility criteria	10	Inclusion and exclusion criteria for participants. If applicable, eligibility criteria for study centres and individuals who will perform the interventions (e.g., surgeons, psychotherapists)	5
Interventions	11a	Interventions for each group with sufficient detail to allow replication, including how and when they will be administered	9, 10, 11
	11b	Criteria for discontinuing or modifying allocated interventions for a given trial participant (e.g., drug dose change in response to harms, participant request, or improving/worsening disease)	10, 11
	11c	Strategies to improve adherence to intervention protocols, and any procedures for monitoring adherence (e.g., drug tablet return, laboratory tests)	15
	11d	Relevant concomitant care and interventions that are permitted or prohibited during the trial	11

Outcomes	12	Primary, secondary, and other outcomes, including the specific measurement variable (e.g., systolic blood pressure), analysis metric (e.g., change from baseline, final value, time to event), method of aggregation (e.g., median, proportion), and time point for each outcome. Explanation of the clinical relevance of chosen efficacy and harm outcomes is strongly recommended	12
Participant timeline	13	Time schedule of enrolment, interventions (including any run-ins and washouts), assessments, and visits for participants. A schematic diagram is highly recommended (see Figure)	6,7 (Table 1)
Sample size	14	Estimated number of participants needed to achieve study objectives and how it was determined, including clinical and statistical assumptions supporting any sample size calculations	11
Recruitment	15	Strategies for achieving adequate participant enrolment to reach target sample size	15
**Methods: Assignment of interventions (for controlled trials)**			
Allocation:			
Sequence generation	16a	Method of generating the allocation sequence (e.g., computer-generated random numbers), and list of any factors for stratification. To reduce predictability of a random sequence, details of any planned restriction (e.g., blocking) should be provided in a separate document that is unavailable to those who enrol participants or assign interventions	Permuted Block randomization, 5
Allocation concealment mechanism	16b	Mechanism of implementing the allocation sequence (e.g., central telephone; sequentially numbered, opaque, sealed envelopes), describing any steps to conceal the sequence until interventions are assigned	Sequentially numbered, opaque, sealed envelopes (Page 5)

Implementation	16c	Who will generate the allocation sequence, who will enrol participants, and who will assign participants to interventions	Allocation sequence- Senior scientist not related to the study. Enrolment and assignment-PI & Study doctors (Page 5)
Blinding (masking)	17a	Who will be blinded after assignment to interventions (e.g., trial participants, care providers, outcome assessors, data analysts), and how	Open-labelled trial
	17b	If blinded, circumstances under which unblinding is permissible, and procedure for revealing a participant's allocated intervention during the trial	N/A
**Methods: Data collection, management, and analysis**			
Data collection methods	18a	Plans for assessment and collection of outcome, baseline, and other trial data, including any related processes to promote data quality (e.g., duplicate measurements, training of assessors) and a description of study instruments (e.g., questionnaires, laboratory tests) along with their reliability and validity, if known. Reference to where data collection forms can be found, if not in the protocol	7, 8
	18b	Plans to promote participant retention and complete follow-up, including list of any outcome data to be collected for participants who discontinue or deviate from intervention protocols	15
Data management	19	Plans for data entry, coding, security, and storage, including any related processes to promote data quality (e.g., double data entry; range checks for data values). Reference to where details of data management procedures can be found, if not in the protocol	12

Statistical methods	20a	Statistical methods for analysing primary and secondary outcomes. Reference to where other details of the statistical analysis plan can be found, if not in the protocol	12
	20b	Methods for any additional analyses (e.g., subgroup and adjusted analyses)	-
	20c	Definition of analysis population relating to protocol non-adherence (e.g., as randomised analysis), and any statistical methods to handle missing data (e.g., multiple imputation)	-
**Methods: Monitoring**			
Data monitoring	21a	Composition of data monitoring committee (DMC); summary of its role and reporting structure; statement of whether it is independent from the sponsor and competing interests; and reference to where further details about its charter can be found, if not in the protocol. Alternatively, an explanation of why a DMC is not needed	14, 15
	21b	Description of any interim analyses and stopping guidelines, including who will have access to these interim results and make the final decision to terminate the trial	12
Harms	22	Plans for collecting, assessing, reporting, and managing solicited and spontaneously reported adverse events and other unintended effects of trial interventions or trial conduct	12
Auditing	23	Frequency and procedures for auditing trial conduct, if any, and whether the process will be independent from investigators and the sponsor	14, 15
**Ethics and dissemination**			
Research ethics approval	24	Plans for seeking research ethics committee/institutional review board (REC/IRB) approval	14

Protocol amendments	25	Plans for communicating important protocol modifications (e.g., changes to eligibility criteria, outcomes, analyses) to relevant parties (e.g., investigators, REC/IRBs, trial participants, trial registries, journals, regulators)	15
Consent or assent	26a	Who will obtain informed consent or assent from potential trial participants or authorised surrogates, and how (see Item 32)	PI or his/her representative Study doctor (Page 8)
	26b	Additional consent provisions for collection and use of participant data and biological specimens in ancillary studies, if applicable	N/A
Confidentiality	27	How personal information about potential and enrolled participants will be collected, shared, and maintained in order to protect confidentiality before, during, and after the trial	7
Declaration of interests	28	Financial and other competing interests for principal investigators for the overall trial and each study site	16
Access to data	29	Statement of who will have access to the final trial dataset, and disclosure of contractual agreements that limit such access for investigators	The PI will have the access to final data set
Ancillary and post-trial care	30	Provisions, if any, for ancillary and post-trial care, and for compensation to those who suffer harm from trial participation	N/A
Dissemination policy	31a	Plans for investigators and sponsor to communicate trial results to participants, healthcare professionals, the public, and other relevant groups (e.g., via publication, reporting in results databases, or other data sharing arrangements), including any publication restrictions	2,15
	31b	Authorship eligibility guidelines and any intended use of professional writers	NA

For peer review only

	31c	Plans, if any, for granting public access to the full protocol, participant-level dataset, and statistical code	
**Appendices**			
Informed consent materials	32	Model consent form and other related documentation given to participants and authorised surrogates	Attached
Biological specimens	33	Plans for collection, laboratory evaluation, and storage of biological specimens for genetic or molecular analysis in the current trial and for future use in ancillary studies, if applicable	7

For peer review only

BMJ Open

Efficacy of dopamine, adrenaline and blood transfusion for treatment of fluid refractory shock in children with severe acute malnutrition or severe underweight and cholera or other dehydrating diarrhoeas: protocol for a randomized controlled clinical trial

Journal:	*BMJ Open*
Manuscript ID	bmjopen-2022-068660.R1
Article Type:	Protocol
Date Submitted by the Author:	20-Jan-2023

Complete List of Authors:	Sarmin, Monira ; International Centre for Diarrhoeal Disease Research Bangladesh, Nutrition and Clinical Services Division<br>Shaly, Nusrat ; International Centre for Diarrhoeal Disease Research Bangladesh, Nutrition and Clinical Services Division<br>Sultana, Tania; International Centre for Diarrhoeal Disease Research Bangladesh, Nutrition and Clinical Services Division<br>Tariqujjaman, Md. ; International Centre for Diarrhoeal Disease Research Bangladesh<br>Shikha, Shamima ; International Centre for Diarrhoeal Disease Research Bangladesh, Nutrition and Clinical Services Division<br>Mariam, Nafisa; International Centre for Diarrhoeal Disease Research Bangladesh, Nutrition and Clinical Services Division<br>Jeorge, Didarul ; International Centre for Diarrhoeal Disease Research Bangladesh, Nutrition and Clinical Services Division<br>Tabassum, Mosharrat; International Centre for Diarrhoeal Disease Research Bangladesh, Nutrition and Clinical Services Division<br>Nahar, Baitun; International Centre for Diarrhoeal Disease Research Bangladesh<br>Afroze, Farzana; International Centre for Diarrhoeal Disease Research Bangladesh<br>Shahrin, Lubaba; International Centre for Diarrhoeal Disease Research Bangladesh, NCSD<br>Hossain, Md. Iqbal ; International Centre for Diarrhoeal Disease Research Bangladesh, Nutrition and Clinical Services Division<br>Alam, Baharul; International Centre for Diarrhoeal Disease Research Bangladesh, Nutrition and Clinical Services Division<br>Faruque, ASG; International Centre for Diarrhoeal Disease Research, Nutrition and Clinical Services Division<br>Islam, M; International Centre for Diarrhoeal Disease Research Bangladesh, Nutrition and Clinical Services Division<br>Osmany, Mohammad ; Bangabandhu Sheikh Mujib Medical University, Department of Cardiology<br>Chowdhury, Meshkat A.; Bangabandhu Sheikh Mujib Medical University, Department of Cardiology<br>Manji, Karim; Muhibili University of Health and Allied Sciences, Department of Pediatrics and Child Health<br>Kissoon, Niranjan; University of British Columbia, Department of

	Pediatrics<br>Chisti, Md.; International Centre for Diarrhoeal Disease Research Bangladesh, Nutrition and Clinical Services Division<br>Ahmed, Tahmeed; International Centre for Diarrhoeal Disease Research Bangladesh, Nutrition and Clinical Services Division
**Primary Subject Heading**:	Paediatrics
Secondary Subject Heading:	Gastroenterology and hepatology, Intensive care, Infectious diseases, Paediatrics
Keywords:	Paediatric intensive & critical care < ANAESTHETICS, Paediatric gastroenterology < PAEDIATRICS, Respiratory infections < THORACIC MEDICINE

SCHOLARONE™
Manuscripts

Title: Efficacy of dopamine, adrenaline and blood transfusion for treatment of fluid refractory shock in children with severe acute malnutrition or severe underweight and cholera or other dehydrating diarrhoeas: protocol for a randomized controlled clinical trial

Short Title: Efficacy of dopamine, adrenaline and blood transfusion for treatment of fluid refractory shock in severe malnutrition

Authors:

Monira Sarmin[1], Nusrat Jahan Shaly[1], Tania Sultana[1], Md Tariqujjaman[1], Shamima Sharmin Shikha[1], Nafisa Mariam[1], Didarul Haque Jeorge[1], Mosharrat Tabassum[1], Baitun Nahar[1], Farzana Afroze[1], Lubaba Shahrin[1], Md. Iqbal Hossain[1], Baharul Alam[1], ASG Faruque[1], Md. Munirul Islam[1], Din-E-Mujahid Mohammad Faruqe Osmany[2], Chowdhury Meshkat Ahmed[2], Karim P Manji[3], Niranjan Kissoon[4], Mohammod Jobayer Chisti[1], Tahmeed Ahmed[1]

Affiliations:

[1]Nutrition and Clinical Services Division, icddr,b, Dhaka, Bangladesh

[2]Department of Cardiology, Bangabandhu Sheikh Mujib Medical University (BSMMU), Dhaka, Bangladesh

[3]Department of Paediatrics and Child Health, Muhimbili University of Health and Allied Sciences, Dar es Salaam, Tanzania

[4]Department of Paediatrics, University of British Colombia & British Colombia Children's Hospital, Canada

Corresponding author: Mohammod Jobayer Chisti (chisti@icddrb.org)

For peer review only - http://bmjopen.bmj.com/site/about/guidelines.xhtml

ABSTRACT

Introduction Diarrhoea is one of the leading causes of under-five childhood mortality and accounts for 8% of 5.4 million global under-5 deaths. In severely malnourished children, diarrrhoea progresses to shock, where the risk of mortality is even higher. At icddr,b Dhaka Hospital, the fatality rate is as high as 69% in children with severe malnutrition and fluid refractory septic shock. To date, no study has evaluated systematically the effects of inotrope or vasopressor or blood transfusion in children with dehydrating diarrhoea (for example, in cholera) and severe acute malnutrition (SAM) or severe underweight who are in shock and unresponsive to WHO-recommended fluid therapy. To reduce the mortality of severely malnourished children presenting with diarrhea and fluid refractory shock, we aim to compare the efficacy of blood transfusion, dopamine, and adrenaline in fluid refractory shock in children who do not respond to WHO-recommended fluid resuscitation.

Methods and analysis In this randomized, three-arm, controlled, non-masked clinical trial in children 1-59 months old with SAM or severe underweight and fluid refractory shock, we will compare the efficacy of dopamine or adrenaline administration versus blood transfusion in children who failed to respond to WHO-recommended fluid resuscitation. The primary outcome variable is the case fatality rate. The effect of the intervention will be assessed by performing an intention-to-treat analysis. Recruitment and data collection began in July 2021 and are now ongoing. Results are expected by May 2023.

Ethics and dissemination This study has been approved by the icddr,b Institutional Review Board. We adhere to the 'Declaration of Helsinki' (2000), guidelines for Good Clinical Practice. Before enrolment, we are collecting signed informed consent from the parents or caregivers of the children. We will publish the results in a peer-reviewed journal and will arrange a dissemination seminar.

Trial registration number ClinicalTrials.gov**;** NCT04750070, February 11, 2021

Version no:2.2, Dated July 21, 2022

For peer review only - http://bmjopen.bmj.com/site/about/guidelines.xhtml

STRENGTHS AND LIMITATIONS OF THIS STUDY

⇒This study includes severely malnourished children with diarrhoea having fluid refractory shock

⇒This study evaluates cardiac function of the study children at bedside by point-of-care (POC) cardiac USG

⇒ This study is excluding children having congenital heart diseases, Down's syndrome or any chronic illness

⇒ Evaluation of non-diarrheal children having shock is out of scope for this study

For peer review only

INTRODUCTION

Background and rationale

Diarrheal diseases are the second leading cause of death in under-five children and were responsible for the deaths of 370,000 children in 2019 [1]. In Bangladesh, diarrhea causes 7.3% of under-5 deaths in 2019[2]. Children with severe acute malnutrition (SAM) frequently present with diarrhea, and fluid refractory shock is one of the common complications of diarrhea [3]. The management of these children is challenging. There is a lack of evidence-based guidelines for the management of fluid refractory shock in severely malnourished children. Optimized management of the altered hemodynamics in fluid refractory shock is yet to be explored and different opinions exist among experts, especially for malnourished children. Data from the Dhaka Hospital of icddr,b demonstrates that despite appropriate care, 69% of children having co-morbidities such as severe malnutrition die from fluid refractory shock [4].

The World Health Organization (WHO) recommends administration of isotonic saline boluses in a dose of 15 mL/kg over 1 hour (maximum two times), followed by blood transfusion in a dose of 10 mL/kg over 3 hours in cases unresponsive to isotonic fluid boluses and have a fluid refractory/septic shock [5]. Quick arrangement of safe blood is difficult, especially in resource-poor settings. The FEAST trial showed significantly higher mortality associated with the administration of saline bolus or albumin [6] in children having severe sepsis. Inotropes like dopamine perform better than excessive fluid administration [7] when myocardial dysfunction or vasoregulatory failure results in shock rather than absolute hypovolemia [7], [8]. Recent studies also demonstrated that adrenaline resulted in the early resolution of shock with survival benefits than dopamine [9], [10]. It is a complex situation whether to use blood or inotropes in fluid refractory shock. Severely malnourished children are in reductive adaptation with

For peer review only - http://bmjopen.bmj.com/site/about/guidelines.xhtml

different hemodynamics than well-nourished children. Despite the very high death rates, the management of fluid refractory shock in severely malnourished children with early inotropes or vasopressors has never been studied.

Malnourished children have less left ventricular mass [11], [12] as well as left ventricular function as evidenced by echocardiography [11], [13] which may constitute a bad prognostic parameter. The FEAST trial reported that in the bolus group a significantly higher proportion had a terminal event cardiac rather than respiratory or neurological [14]. Point of care (POC) cardiac ultrasound is an emerging tool to assess cardiac function and fluid responsiveness in shock and it is non-invasive. The use of the POC ultrasound to differentiate hypovolemic shock from distributive shock e.g. fluid refractory/septic shock, by measuring ventricular function and IVC collapsibility can reduce the diagnostic time as well as increase the accuracy of diagnosis [15], [16]. Usually, in hypovolemic shock, both end-diastolic and end-systolic volume are reduced [16], left ventricle (LV) is small having a hyperdynamic wall motion [17]. Whereas in fluid refractory/septic shock, LV is hyperdynamic [17] and only end-systolic volume is reduced [16]. Again, the diameter of the IVC at inspiration and expiration and their ratio provides useful information regarding fluid responsiveness [16]. To date, no study has evaluated systematically the effects of vasopressor and inotrope(s) in children with dehydrating diarrhea (for example, in cholera) and SAM having fluid refractory shock unresponsive to conventional WHO-recommended fluid therapy.
The proposed study will provide valuable data to determine whether resuscitation strategies including vasopressor and inotropes or blood results in increased survival in children who are severely malnourished having fluid refractory shock and diarrhea. In addition, POC cardiac ultrasound will provide information on whether the shock resulted from a fluid deficit or cardiac dysfunction.

Objective

The study aims to compare the efficacy of blood transfusion, dopamine, and adrenaline in fluid refractory shock in children having SAM or severe underweight who do not respond to WHO-recommended fluid resuscitation.

Trial design

This is a randomized, three-arm, controlled, non-masked clinical trial in children 1-59 months old with SAM or severely underweight and shock (Figure 1). With parental written informed consent, children are allocated to the study interventions following randomization, in addition to usual supportive care that includes broad-spectrum antibiotics, oxygen inhalation, IV glucose, etc. The study

For peer review only - http://bmjopen.bmj.com/site/about/guidelines.xhtml

protocol follows the Standard Protocol Items: Recommendations for Interventional Trials (SPIRIT) guidance for protocol reporting (modified to reflect the ongoing study activities) [18] (Table 1).

METHODS

Participants and interventions and outcomes

Study setting

The study is being conducted at the Dhaka Hospital of the icddr,b, Dhaka, Bangladesh. icddr,b is the largest diarrheal diseases hospital in the world. Annually a total of 194,838 patients of all ages and both genders got treatment and care from icddr,b, and 57.2% of them were under five years of age. Besides diarrheal illness, many patients have other health conditions, like pneumonia, malnutrition, sepsis, and electrolyte abnormalities. For critically ill patients, there is an intensive care unit facility present within the hospital, equipped with contemporary life support measures, including mechanical ventilators, bubble continuous positive airway pressure (BCPAP) oxygen therapy, high flow nasal cannula (HNFC) oxygen therapy, and syringe pumps for vasopressor support. The ICU of the Dhaka Hospital of icddr,b manages around 1000 pediatric patients each year. More than 80% of them have a poor socio-economic background. About one-third have severe malnutrition, and one-fourth of them have severe sepsis or fluid refractory shock, there are other different wards for the treatment of diarrheal illness, respiratory problems, and malnutrition. icddr,b possesses a well-equipped laboratory service capable of performing a range of clinical tests proposed in this study.

Inclusion criteria are children aged 1-59 months of either sex having diarrhea and severe acute malnutrition who develop fluid refractory shock either at admission or at any point during their treatment in the hospital (which might be due to cholera or other dehydrating diarrhea or severe sepsis). We also included children having severe underweight as they are also vulnerable and their mortality rate is also high.

Exclusion criteria are severe anaemia (hemoglobin < 5 gm/dl) and sclerema where blood transfusion is a mandate, congenital anomalies (tetralogy of Fallot (TOF)/atrial or ventricular septal defect (ASD or VSD)/Trisomy 21, etc.), negative blood groups, a requirement of cardio-pulmonary resuscitation during screening and non-consent from the caregivers/parents.

For peer review only - http://bmjopen.bmj.com/site/about/guidelines.xhtml

Randomization: The permuted block randomization technique is followed to select treatment arms for each child. The randomization procedure was planned and executed by a scientist from icddr,b who is not involved in the study. The randomization list containing the subject identification numbers (IDs) and the corresponding group allocation remained concealed. IDs are chronologically assigned to each new study participant. After randomization, an opaque envelope containing the name of the allocated intervention group is opened.

Study duration: Twenty-four months

For peer review only

For peer review only - http://bmjopen.bmj.com/site/about/guidelines.xhtml

Table 1: Standard Protocol Items: Recommendation for Interventional Trials (SPIRIT) Figure. The schedule of enrolment, interventions, and assessments

**Indicators**		**Study period**						
	Screening	Enrolment & allocation	Study drugs administration & F-U	Rescue therapy	Hosp course up to disposal	Follow up after discharge		
						1st follow up	2nd follow up	28th day follow up (over phone)
Time point	Day 0	Hour 0	Hour 0-3 (1 hour for Dopamine & Adrenaline, 3 hours for Blood)	Hour 1- disposal (for Dopamine & Adrenaline) Hour 3- disposal (for Blood)		Day 7±2	Day 21±2	Day 28±3
Enrolment								
Eligibility screen	x							
Informed consent	x	x						
Randomization and allocation		x	x					
Interventions								
Different drugs (Dopamine or Adrenaline or Blood)			x					
Assessments:								
Demographic		x						
Comorbidities		x						
Physical examination	x	x	x	x	x	x	x	-
Primary outcome:								
case fatality								x
Secondary outcomes:								
Treatment failure			x					
MAP stabilization at 48 hours after enrolment				x	x			
Mechanical ventilation			x	x	x			

For peer review only - http://bmjopen.bmj.com/site/about/guidelines.xhtml

Incidence of heart failure			x	x	x			
Duration of ICU stay					x			
Duration of total hospitalization					x			
Time-to-achieve recovery from shock			x	x	x			
Ventricular function by cardiac USG	x	x		x	x (if require)			
**IVC (Inferior Vena Cava) collapsibility**	x	x		x	x (if require)			
[a]Detection of leptin from stored blood		x						

[a]According to the addendum (ERC approval on October 21 2021), at the end of the study, stored blood samples will be tested for leptin.

Collection of Baseline Information

Any SAM children with fluid refractory shock, either at admission or developed at some point during their treatment period, within the defined age group are screened for study eligibility criteria by the study physician. According to the inclusion and exclusion criteria, parents or attending caregivers of those eligible children are invited for providing consent for their children to be enrolled in the study. A signed written informed consent form by parents or caregivers gives data concerning the study, attainable advantages and risks, and voluntary nature of participation, as well as the right to cancel the child's participation in the study at any time (even after giving the initial consent) while not providing any reason; after this, children are enrolled by the study physician. One copy of the signed consent document was given to the caregiver of every participant and another copy was reserved for the study documentation. A pretested case record form is used to collect relevant medical history information, including the nature and duration of illness, medication for current illness, and any history of significant past illness including pneumonia and diarrhea. The form is also used to collect information on sociodemographic characteristics, such as age, sex, religion, gestational age, parental age with education and occupations, source of drinking water and sanitation, type of fuel use, cooking place and smoking history, average monthly income of the family, number of siblings, and number of rooms in the home. Information is also collected about each child's feeding practice, which included their history of breastfeeding, formula feedings, or alternative complementary feedings, and also immunization status. Data on clinical examination findings are recorded by the study physician and include pulse, respiratory rate, axillary temperature, anthropometric measurements (ie, height, weight, mid-upper arm

For peer review only - http://bmjopen.bmj.com/site/about/guidelines.xhtml

circumference for calculation of Z score), chest auscultation, oxygen saturation, presence or absence of chest wall indrawing, cyanosis, and mental status (ie, normal, irritable, or lethargic), and response according to AVPU. Each child's weight is measured by using an electronic weighing scale with a precision of 0.1 kg; length is measured using a standard-length board with a precision of 0.5 cm and mid-upper arm- circumference (MUAC) is measured using a standard MUAC tape with a precision of 0.2 cm by the trained and experienced study nurse of Dhaka hospital. Fever is defined when the axillary temperature is 38°C or greater. Respiratory rate is counted for a full 60 seconds by exposing the trunk when the child is awake and calm; the presence of lower-chest-wall indrawing is noted at the same time. Oxygen saturation is measured using a portable pulse oximeter (Handheld Vital Signs Monitors- NT1D, Solaris Medical Technology, Inc, USA) with a probe on a finger or toe when the child breathes in room air. Oxygen saturation of <90% is defined as hypoxemia, which is an indication for supplemental oxygen therapy by using BCPAP. Blood group and Rh typing is done at the bedside and we exclude the child having negative blood groups.

Bedside Hb (if not known) is checked by using Hemocue analyser during the screening period to exclude severely anemic children requiring an urgent blood transfusion. The length of ICU stays, the total length of hospitalization and time-to-achieve-recovery (time required for the restoration of MAP after the starting of the intervention for fluid refractory shock) are recorded. We are also keeping a record of stool and urine volume. Any deterioration or adverse events (ie, signs of heart failure or need for mechanical ventilation) are also documented. Both the study and outcome are recorded including treatment failure/success, hospital discharge, referrals, and deaths or left against medical advice (LAMA).

Laboratory Testing and sample handling

We are performing laboratory tests after the enrolment of a child in the study that includes blood for haemoglobin percentage, total and differential counts of white blood cell (WBC), hematocrit, blood culture and antimicrobial susceptibility of pathogens, serum electrolytes and creatinine, calcium and magnesium, serum bilirubin, serum lactate, C-reactive protein (CRP), procalcitonin, and cardiac troponin I, stool (rectal swab for Cholera, Salmonella, Shigella and Campylobacter) and urine culture, chest radiograph, and arterial or capillary blood gas analysis. We are also doing a test for the exclusion of dengue during the dengue prevalent period in the country. We are checking serum lactate at enrolment and 4-6 hours after enrolment. Our target is to send samples for laboratory tests within 30 min-1 hour of enrolment. When a child is in shock, very often it is difficult to get an adequate amount of blood for proposed testing. Then we prioritize tests based on clinical relevance. We are doing the blood grouping, screening and cross-matching while we are following a child for study eligibility. We are performing

For peer review only - http://bmjopen.bmj.com/site/about/guidelines.xhtml

cardiac ultrasonography during screening, enrolment and 4-6 hours later after enrolment. As we are enrolling children 24X7, the study physicians are doing the bedside cardiac ultrasound and storing the images of specific pre-selected parameters. Later the images are reviewed by the experienced cardiologists to get the measurements.

We are archiving blood samples and planning to check serum leptin at the end of the study.

Study intervention and procedure

This is a three-arm trial where the intervention arms (dopamine and adrenaline) will be compared with the WHO standard intervention (blood transfusion). All study children stay at ICU for close monitoring. During the screening, children receive intravenous isotonic fluid @ of 15mL/kg over one hour (maximum two boluses i.e. up to 30ml/kg) as per WHO standard of care. The choice of isotonic fluid is Ringer`s lactate/Hartsol (Normal saline if any suspicion of hyperkelemia, acetate if severe dehydration is predominant). Dextrose is added if a child has hypoglycaemia with a random blood sugar <3 m mol/l. The study team members work to assess the eligibility and discuss with the parents/caregiver for possible enrolment. the regular ICU team helps in patient care. At the end of screening, if fluid refractory shock is diagnosed, then the study team proceed for written consent to avert gap between consent to starting appropriate management of the child.

Children with fluid refractory shock are randomized to blood transfusion, dopamine, or adrenaline therapy. Children in the dopamine arm (**Treatment category A**) receive dopamine, 8 microgram/kg.min (increasing the dose after 15 minutes to 12 microgram/kg/min to a maximum of 15 microgram/kg/min, up to the achievement of goal). Children in the adrenaline arm (**Treatment category B**) receive adrenaline, 0.1 microgram/kg/min (increasing the dose after 15 minutes to 0.2 microgram/kg.min to a maximum of 0.3 microgram/kg.min, up to the achievement of goal). We are using peripheral lines to give dopamine or adrenaline. Drugs are prepared as IV infusion and instilled by an infusion/syringe pump. Children in the blood transfusion arm (**Treatment category C)** receive a transfusion of whole human blood in a dose of 10 mL/kg over 3 hours (Figure 1). To arrange blood within the time frame, we have an institutional volunteer donor list, two medical technologists dedicatedly work for this study, and we are also excluding children having negative blood groups. Even after that, If blood could not be arranged within one hour after the event of fluid refractory shock, we will start rescue therapy considering the event as treatment failure. In addition to the study interventions, broad-spectrum antibiotic therapy, oxygen therapy, and frequent monitoring, all children receive IV maintenance fluids with half-strength normal saline and 5% dextrose @ of 3 ml per kg per hour during the arrangement of blood transfusion and intervention with inotropes. Along with maintenance fluid, we add IV fluid if diarrhoea continues or a child develops

For peer review only - http://bmjopen.bmj.com/site/about/guidelines.xhtml

dehydration. IV fluid maintenance is continued for 6 hours after the achievement of a goal and is further continued if the goal is not achieved. Our goal is to achieve MAP >50 mm of Hg and/or urine output ≥1 ml/kg per hour. After randomization, we monitor children at every 15-minute interval during the intervention to assess mental status by using AVPU, an acronym from "alert, verbal, pain, unresponsive", capillary refill time, rate and volume of the radial pulse, cold/warm extremities, urine output, blood pressure, respiratory rate, peripheral capillary O_2 saturation, and features of heart failure. We measure BP manually using standard cuff. To minimize the use of catheter, we apply a paediatric urine collector bag and measure the urine output. Mechanical ventilation is provided if the child did not respond to bubble CPAP (if SpO2<85% at least after an hour of bubble CPAP oxygen therapy) or had a respiratory failure (Ph<7.2 and PCO_2>60 mm of Hg in ABG analysis or SpO_2/FiO_2<265). Children who develop complications such as acute abdomen not responding to conservative management or acute renal insufficiency requiring dialysis are referred to a suitable higher facility for proper management. At the achievement of the goal, we continue the dose of dopamine or adrenaline for 6 hours. We follow up with the child every 30 min to 1-hour interval. Thereafter we reduce the dose in a stepwise manner similar to how we increased the dose. If the child becomes hypotensive again, we re-introduce the previous dose and follow the same procedure for further iteration.

Rescue therapy

The failure of any intervention is declared as a treatment failure. Then for children in dopamine and blood transfusion arm, we start adrenaline as rescue therapy in a stepwise manner starting from 0.1 microgram/kg/min and increasing every 15 min by 0.1 microgram/kg/min up to restoration of the desired goal (MAP >50 mm of Hg or urine output>1ml/kg/hour) and the highest dose is 0.5 microgram/kg/min (or consultant discretion) For children in the adrenaline arm, the dose is increased every 15 minutes from 0.3 microgram/kg/min (the highest proposed intervention dose) to a max of 0.5 microgram/kg/min. Upon achieving the goal, we continue the same dose for 4-6 hours and then we de-escalate in a stepwise manner.

If the goal is not achieved with injection of adrenaline then injection of noradrenaline is started @ 0.1 microgram/kg/min and we increase the dose every 15 min by 0.1 microgram/kg up to restoration of the desired goal (MAP >50 mm of Hg or urine output>1ml/kg/hour) and the highest dose is 0.5 microgram/kg/min. Upon achieving the goal, we continue the same dose for 6 hours and then will de-escalate in a stepwise manner. For those patients whose MAP is not restored with inotropes, injection hydrocortisone @ 2mg/kg every 6 hours is added [19]. During rescue therapy, for doses and selection of

For peer review only - http://bmjopen.bmj.com/site/about/guidelines.xhtml

any inotrope(s), we are following the recommendation from the PICU consultant as all cases are not the same.

Feeding and other care

Depending on the clinical condition either we keep the child NPO or start trophic feeding for children who had MAP >50 mm of Hg and had no evidence of paralytic ileus. Feeds consist of "Milk Suzi" (similar to WHO recommended F-75) for children older than 6 months, and modified infant formula (in addition to breast milk, if applicable) is given to infants 1-6 months of age [20]. Thus, we provide a fluid volume of 120ml/kg/day to deliver 80-82 Kcal/kg/day. A child is kept warm to prevent hypothermia. If there is hypoglycaemia (random blood glucose < 3mmol/L), correction is given with 10% glucose intravenously in a dose of 5 mL/kg to be followed by 10% glucose administration through a nasogastric tube, following the standard protocol of the hospital.

Nutritional rehabilitation

As the study children have severe acute malnutrition, they require nutritional rehabilitation. After recovering from acute illness, the mothers/caregivers are asked to stay in the hospital Nutritional Rehabilitation Unit (NRU) for 2-3 weeks. In the NRU, the standard dietary protocol is followed for nutritional rehabilitation and the children are discharged upon fulfilling discharge criteria.

Post-discharge follow-up

We follow up with a child on the 28th day (±3 days) of enrolment to know their clinical status over the phone. In addition, the children who were discharged from NRU or taking early discharge from the ward, are requested to come for a pre-scheduled follow-up at the hospital nutrition follow-up unit (NFU) of icddr,b. We followed the children at NFU and record their nutritional status and health problems. Our scheduled follow-ups for this study children are in two-time points; once after 7 days of discharge, then after 15 days of 1st follow-up. If any mother/caregiver fails to attend a follow-up visit, then we communicate with the mother/caregiver over the phone for two-time points. We document the outcome whether they are alive or not, and had any intercurrent illness e.g. diarrhea, pneumonia.

For peer review only - http://bmjopen.bmj.com/site/about/guidelines.xhtml

For peer review only

Outcome measures

Primary outcome variable is the case fatality rate (at 28±3 days from the day of enrolment). The secondary outcome variables are the rate of treatment failure, rate of MAP stabilization at 48 hours after enrolment, need for mechanical ventilation, the incidence of heart failure, duration of ICU stay and total hospitalization, time-to-achieve recovery, evaluation of cardiac function by point of care ultrasonogram (right ventricular function, left ventricular function and Inferior Vena Cava (IVC) collapsibility).

Sample size

The 2010-11 data from the ICU of the Dhaka Hospital of the icddr,b (the proposed study site) revealed that among the 36 severely malnourished children with shock, 22 died i.e. mortality rate among SAM children with septic shock was 61% (22/36). We assumed that the proposed interventions would reduce the case fatality rate by 50%, i.e. the rate will decrease from 61% to 31%.

To detect this magnitude of difference in the death rate, with 80% power (20% type II error) and 0.05 type I error, the required sample size is 40 children in each of the three groups. Considering around 10% dropouts after enrolment, the total sample size would be 45 children at least in each of the three groups, i.e. a total of 135 children - the final sample size of our study.

Data collection, management and analysis

Data will be collected using a hard copy structured case record form . Data will be entered into a personal computer on the online platform, REDCap. The data management team will check the consistency of data and will download and store the dataset as a backup to ensure data security. Statistical analyses will include descriptive as well as analytical methods. The effect of the intervention will be assessed by performing an intention-to-treat analysis as well as a per-protocol analysis.; Statistical analyses will include descriptive as well as analytical methods. We anticipate that there will be no differences in the sociodemographic data among the three groups. Categorical variables will be compared using the chi-square test or Fisher exact test as appropriate. When the variables of interest are continuous and distributed normally , the statistical significance of group means will be evaluated by analysis of variance. When the variables are continuous and not-normally distributed , the statistical significance of group differences will be determined by the Kruskal-Wallis test. The post hoc test will be done if required. The multiple log linear regression analyses will be performed after adjusting for potential confounders including child's age, child's sex, child's morbidity status, wealth status etc to reach more definitive conclusions. The strength of association will be determined by calculating the relative risk (RR)

For peer review only - http://bmjopen.bmj.com/site/about/guidelines.xhtml

and their 95% confidence intervals (CIs). In addition, we will analyze the Kaplan-Meier survival curve to compare the survival times among the three treatment groups. A probability of less than 0.05 will be considered statistically significant. The schedule interim analysis will be done after 50% of enrolment or earlier if DSMB advise. We will follow the stopping rule if the P <0.003, as recommended by Pato and Fleming [21]. Data analysis will be done using SPSS for Windows (Version 20.0. Armonk, NY: IBM Corp and Epi Info (version 7.0, USD, Stone Mountain, GA) or STATA (version 15.0 SE).

Reporting of adverse events

Serious adverse events (deaths) after initiation of the study intervention are recorded and presented to the DSMB within 24 hours of the event. The report includes a detailed clinical history, the study intervention, and management given until the outcome. We contacted over the phone for study participants who are referred.

Operational definition

Severe Acute Malnutrition (SAM): A child with a <-3 Z-score of weight for height/length (WHZ/WLZ) of the median value of the WHO standard or MUAC<115 mm (for children >6 months of age) /visible wasting (for children <6 months of age), or presence of nutritional edema.

Severe underweight: A child with a <-3 Z-score of weight for age (WAZ) of the median value of the WHO standard.

Sepsis: Presence of infection/inflammation plus hyperthermia or hypothermia (temperature >38.5º Celsius or <35.0º Celsius respectively) plus tachycardia (HR: infant>160/min, 1-5 years>140/min) or abnormal WBC count (>12×10⁹/L* or, <4 ×10⁹/L or, band and neutrophil ratio ≥ 0.1)

Severe sepsis: Severe sepsis is defined as the presence of sepsis plus organ dysfunction or tissue hypoperfusion [poor peripheral perfusion (hypotension and/ or absent peripheral pulses and/or delayed capillary refilling time (CRT) in the absence of dehydration or after correction of dehydration)]

Fluid refractory shock or Septic Shock: Shock is defined hypotension (Mean arterial pressure <50mm of Hg) and or reduced urine output <1 mL/kg/h despite fluid resuscitation [22]. This 50 mm Hg cut off was fixed on the basis of our local evidence from the same population [22]. A child in septic shock usually has cold periphery, delayed CRT, low volume or absent pulse.

For peer review only - http://bmjopen.bmj.com/site/about/guidelines.xhtml

Mean arterial pressure (MAP): MAP is calculated from systolic blood pressure (SBP) and diastolic blood pressure (DBP) by using a formula: DBP added to 1/3rd of pulse pressure (subtraction of the DBP from the SBP).

Treatment failure: Treatment failure is defined if an intervention results in

- ☐ MAP < 50 mm of Hg or
- ☐ Urine output is < 1 ml/kg per hour, at the end of intended intervention over 2/3 hour

Heart failure (a combination of findings)

- ☐ Age-specific tachypnea
- ☐ Tachycardia
- ☐ Enlarged tender liver
- ☐ Pedal edema (new or worsening)
- ☐ Basal crackles and/or
- ☐ Gallop and
- ☐ Response to furosemide

Signs of improvement (during 1st hour/2nd hour of fluid bolus therapy)

- ☐ Pulse rate falls
- ☐ Pulse volume improves
- ☐ Respiratory rate falls
- ☐ No signs of pulmonary edema

Signs of deterioration (during 1st hour/2nd hour of fluid bolus therapy)

- ☐ Breathing rate increases by 5/minute
- ☐ Pulse rate increases by 15/minute
- ☐ Liver enlarges (by measuring size at the beginning of fluid resuscitation and at the time of deterioration)
- ☐ Fine crackles at the lung base or throughout the lung fields
- ☐ Edema (new or worsening)
- ☐ Galloping heart rhythm

Acute kidney injury (AKI): AKI is defined as an increase in serum creatinine by ≥ 0.3 mg/dL from baseline within 48 hours OR, an increase in serum creatinine to ≥ 1.5 times baseline or age-specific upper limit of reference interval (ULRI) OR, urine volume ≤ 0.5 mL/kg/hour for six hours.

Recovery time: Time required for the restoration of MAP after starting the intervention for shock

Congenital heart diseases: Atrial septal defect, ventricular septal defect, Tetralogy of Fallot (TOF)

Organic/other diseases: Down`s syndrome, cerebral palsy

For peer review only

Patient and public involvement

We have planned to conduct public engagement activities like round table discussions and workshops at the end of the study.

Ethics

This study has been approved by the Institutional Review Board (IRB) of the icddr,b (Protocol No. PR#20021, version 2.2, dated 21.07.2022). The study is performing in compliance with the 'Declaration of Helsinki' (2000), Guideline for Good Clinical Practice (GCP). Before enrolment, we are collecting signed informed consent from the parents or caregivers of the children. We are strictly maintaining privacy, anonymity, and confidentiality of data or information identifying the patients or caregivers. Participants' medical information, particulars of treatment, and laboratory test results will be kept confidential and under lock and key; only the research staff will have access to this information. The risk to enrolled subjects from participation in the study is minimal. Adverse medical events are anticipated as the study is enrolling critically ill patients. This protocol has been reviewed & approved by the Data Safety and Monitoring Board (DSMB) of icddr,b. DSMB is a five membered board who independently review the activity of study. We are reporting the occurrence of any serious adverse event (e.g., death) to the DSMB within 24 hours of the event and will follow their recommendations. There is no influence of the funder at any stage of the research, starting from study design and up to publication. Study patients are recruited into three different groups. For this clinical trial, inj adrenaline and inj dopamine are being used in fluid refractory shock. These drugs are already in use in the ICU of Dhaka hospital of icddr,b [22]. WHO recommends blood transfusion, and we are routinely practicing it. Regardless of the study result, finally, it will be disseminated.

Strategies for achieving adequate participant enrolment

Treatment at icddr,b Dhaka Hospital is free of cost for all patients. In addition to routine care, the study children get more follow up by study physicians. So, they get the benefit of early detection and early intervention for any clinical condition. The caregiver has access to the study investigators and other physicians to communicate and to get an answer to their questions related to their child's medical condition and treatment even after their discharge. However, we pay a small amount of money (transport cost) for their follow-up visit after their usual discharge. We believe this information and empathetic care from the study will ensure adequate participant enrolment to reach the target sample size and also reduce the number of lost to follow up.

For peer review only - http://bmjopen.bmj.com/site/about/guidelines.xhtml

Dissemination

After approval from the investigators of the protocol, we will publish the results and other findings of the study. We will use The International Committee of Medical Journal Editors guidelines to ascertain the authorship of papers.

Study status: As of August 2022, we have screened 49 children and enrolled 18 participants. Participants screening and enrolment are ongoing.

Protocol amendments

After the first approval of the protocol, we included two cardiologists as co-investigators. After one year of enrolment we made change in the inclusion criteria also. Now we are including severe underweight children along with SAM children. So, we required amendments of the protocol which were all reviewed and approved by the icddr,b IRB. Now we are using version 2.2 of the protocol.

Contributorship statement

Monira Sarmin (MS), Nusrat Jahan Shaly (NJS), Tania Sultana (TS), Md Tariqujjaman (MT), Shamima Sharmin Shikha (SSS), Nafisa Mariam (NM), Didarul Haque Jeorge (DHJ), Mosharrat Tabassum (MT), Baitun Nahar (BN), Farzana Afroze (FA), Lubaba Shahrin (LS), Md. Iqbal Hossain (MIH), Baharul Alam (BA), ASG Faruque (ASGF), Md. Munirul Islam (MMI), Din-E-Mujahid Mohammad Faruqe Osmany (DEMMFO), Chowdhury Meshkat Ahmed (CMA), Karim P Manji (KPM), Niranjan Kissoon (NK), Mohammod Jobayer Chisti (MJC), Tahmeed Ahmed (TA)

The study concept and design were conceived by MS, BN, LS, FA, MIH, BA, ASGF, MMI, DEMMFO, CMA, KPM, NK, MJC, and TA. NJS, TA, FA, LS, SSS, NM, DHJ, MT, MJC, and MS are conducting patient screening, patient management, and data collection. Data management is performed by MT, NJS, TA, FA, SSS, NM, DHJ, MT, and MS . Analysis will be performed by MT, MS, MJC, LS, and TA. MS, LS, NJS, TS, MT and MJC prepared the first draft of the manuscript. All authors reviewed, edited and critiqued the manuscript.

Competing interest

None declared

Funding

This protocol is supported by Wellcome Trust, Grant number- 215691/Z/19/Z.

For peer review only - http://bmjopen.bmj.com/site/about/guidelines.xhtml

Acknowledgement

icddr,b is grateful to the governments of Bangladesh, Canada, Sweden and the UK for providing core/unrestricted support.

References

1. WHO. Diarrhoea. 2022
2. Unicef. Diarrhoea 2022 [Available from: https://data.unicef.org/topic/child-health/diarrhoeal-disease accessed 26 April 2022].
3. Chisti MJ, Pietroni MA, Smith JH, et al. Predictors of death in under-five children with diarrhoea admitted to a critical care ward in an urban hospital in Bangladesh. *Acta paediatrica* 2011;100(12):e275-e79.
4. Chisti MJ, Salam MA, Bardhan PK, et al. Severe sepsis in severely malnourished young Bangladeshi children with pneumonia: A retrospective case control study. *PloS one* 2015;10(10):e0139966.
5. Organization WH. Pocket book of hospital care for children: guidelines for the management of common childhood illnesses: World Health Organization 2013.
6. Maitland K, Kiguli S, Opoka RO, et al. Mortality after fluid bolus in African children with severe infection. *New England Journal of Medicine* 2011;364(26):2483-95.
7. Brierley J, Carcillo JA, Choong K, et al. Clinical practice parameters for hemodynamic support of pediatric and neonatal septic shock: 2007 update from the American College of Critical Care Medicine. *Critical care medicine* 2009;37(2):666.
8. Gill A, Weindling A. Randomised controlled trial of plasma protein fraction versus dopamine in hypotensive very low birthweight infants. *Archives of disease in childhood* 1993;69(3 Spec No):284-87.
9. Ventura AM, Shieh HH, Bousso A, et al. Double-Blind Prospective Randomized Controlled Trial of Dopamine Versus Epinephrine as First-Line Vasoactive Drugs in Pediatric Septic Shock. *Critical care medicine* 2015;43(11):2292-302. doi: 10.1097/ccm.0000000000001260 [published Online First: 2015/09/01]
10. Ramaswamy KN, Singhi S, Jayashree M, et al. Double-Blind Randomized Clinical Trial Comparing Dopamine and Epinephrine in Pediatric Fluid-Refractory Hypotensive Septic Shock. *Pediatr Crit Care Med* 2016;17(11):e502-e12. doi: 10.1097/pcc.0000000000000954 [published Online First: 2016/09/28]
11. ABUFADDAN N, Khalid E, Shams H, et al. Myocardial dysfunction in malnourished children. *Journal of Pediatric Sciences* 2010;2(2)
12. Olivares JL, Vazquez M, Rodríguez G, et al. Electrocardiographic and echocardiographic findings in malnourished children. *Journal of the American College of Nutrition* 2005;24(1):38-43.
13. El-Sayed H, Nassar M, Habib N, et al. Structural and functional affection of the heart in protein energy malnutrition patients on admission and after nutritional recovery. *European journal of clinical nutrition* 2006;60(4):502.

For peer review only - http://bmjopen.bmj.com/site/about/guidelines.xhtml

14. Maitland K, George EC, Evans JA, et al. Exploring mechanisms of excess mortality with early fluid resuscitation: insights from the FEAST trial. *BMC Med* 2013;11:68. doi: 10.1186/1741-7015-11-68 [published Online First: 2013/03/19]
15. Jones AE, Tayal VS, Sullivan DM, et al. Randomized, controlled trial of immediate versus delayed goal-directed ultrasound to identify the cause of nontraumatic hypotension in emergency department patients. *Critical care medicine* 2004;32(8):1703-8. doi: 10.1097/01.ccm.0000133017.34137.82 [published Online First: 2004/08/03]
16. Sun J-T. New Advances in Emergency Ultrasound Protocols for Shock. *J Med Ultrasound* 2017;25(4):191-94. doi: 10.1016/j.jmu.2017.09.005 [published Online First: 12/06]
17. Atkinson PRT, McAuley DJ, Kendall RJ, et al. Abdominal and Cardiac Evaluation with Sonography in Shock (ACES): an approach by emergency physicians for the use of ultrasound in patients with undifferentiated hypotension. *Emergency Medicine Journal* 2009;26(2):87-91. doi: 10.1136/emj.2007.056242
18. Chan A, Tetzlaff J, Altman D, et al. Krle a-Jeri K, et al. SPIRIT 2013 statement: defining standard protocol items for clinical trials. *Annals of Internal Medicine* 2013;158(3):200-7.
19. Weiss SL, Peters MJ, Alhazzani W, et al. Surviving sepsis campaign international guidelines for the management of septic shock and sepsis-associated organ dysfunction in children. *Intensive care medicine* 2020;46(1):10-67.
20. Ahmed T, Ali M, Ullah MM, et al. Mortality in severely malnourished children with diarrhoea and use of a standardised management protocol. *The Lancet* 1999;353(9168):1919-22.
21. Science PWCo. Frequentist Methods: O'Brien-Fleming, Pocock, Haybittle-Peto [Available from: https://online.stat.psu.edu/stat509/node/80/.

22.Sarmin M, Ahmed T, Bardhan PK, et al. Specialist hospital study shows that septic shock and drowsiness predict mortality in children under five with diarrhoea. *Acta Paediatr* 2014;103(7):e306-11. doi: 10.1111/apa.12640 [published Online First: 2014/03/26]

Figure 1

Study flow chart showing the procedures of screening and enrolment and allocation of a child in any of the three treatment categories and ways of rescue therapy if the study goal is not achieved

For peer review only - http://bmjopen.bmj.com/site/about/guidelines.xhtml

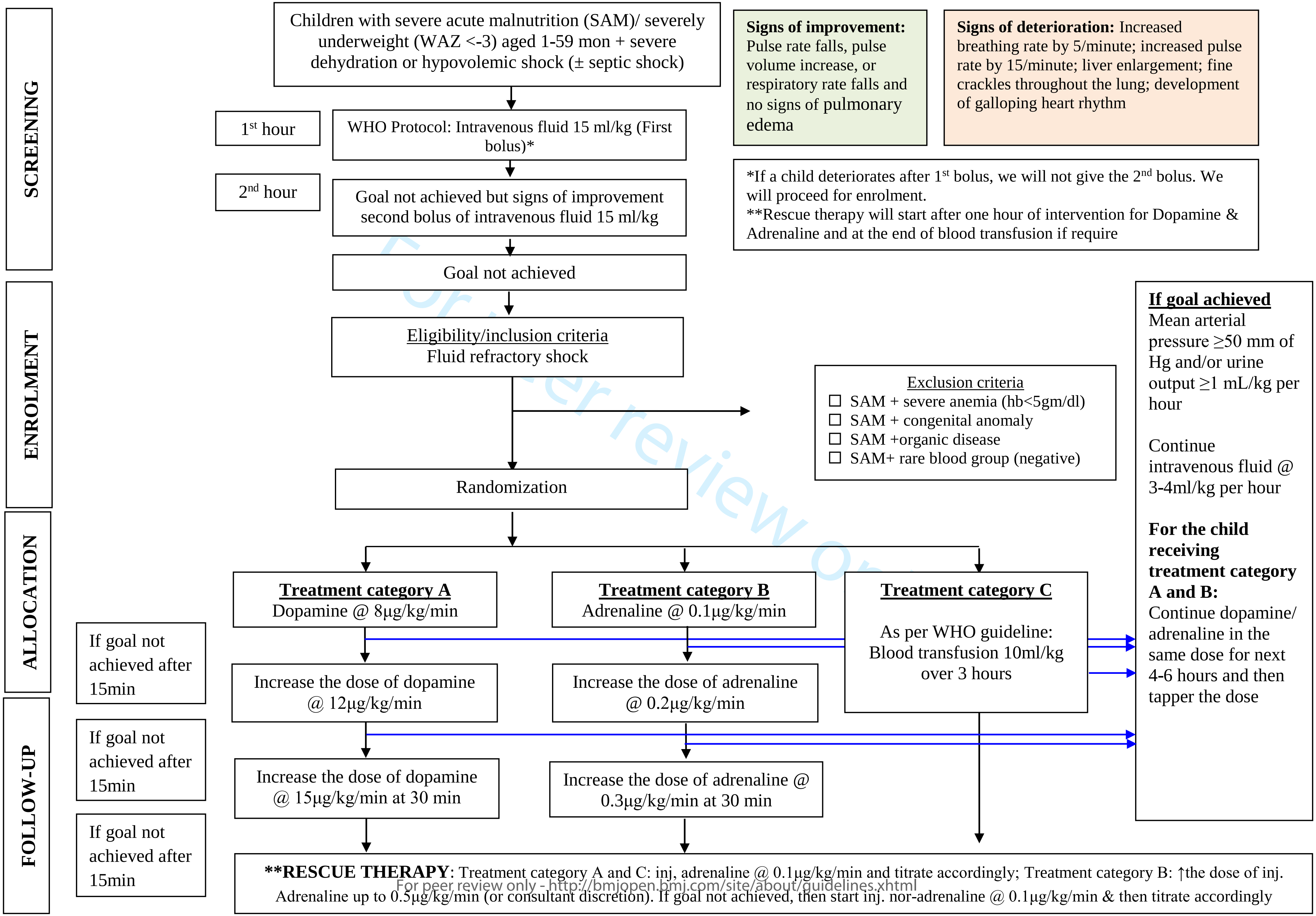
SCREENING
ENROLMENT
ALLOCATION
FOLLOW-UP
Children with severe acute malnutrition (SAM)/ severely underweight (WAZ <-3) aged 1-59 mon + severe dehydration or hypovolemic shock (± septic shock)
1st hour
WHO Protocol: Intravenous fluid 15 ml/kg (First bolus)*
2nd hour
Goal not achieved but signs of improvement second bolus of intravenous fluid 15 ml/kg
Goal not achieved
Eligibility/inclusion criteria
Fluid refractory shock
Randomization
Signs of improvement: Pulse rate falls, pulse volume increase, or respiratory rate falls and no signs of pulmonary edema
Signs of deterioration: Increased breathing rate by 5/minute; increased pulse rate by 15/minute; liver enlargement; fine crackles throughout the lung; development of galloping heart rhythm
*If a child deteriorates after 1st bolus, we will not give the 2nd bolus. We will proceed for enrolment.
**Rescue therapy will start after one hour of intervention for Dopamine & Adrenaline and at the end of blood transfusion if require
Exclusion criteria
☐ SAM + severe anemia (hb<5gm/dl)
☐ SAM + congenital anomaly
☐ SAM +organic disease
☐ SAM+ rare blood group (negative)
If goal achieved
Mean arterial pressure ≥50 mm of Hg and/or urine output ≥1 mL/kg per hour
Continue intravenous fluid @ 3-4ml/kg per hour
For the child receiving treatment category A and B:
Continue dopamine/ adrenaline in the same dose for next 4-6 hours and then tapper the dose
Treatment category A
Dopamine @ 8µg/kg/min
Treatment category B
Adrenaline @ 0.1µg/kg/min
Treatment category C
As per WHO guideline: Blood transfusion 10ml/kg over 3 hours
If goal not achieved after 15min
Increase the dose of dopamine @ 12µg/kg/min
Increase the dose of adrenaline @ 0.2µg/kg/min
If goal not achieved after 15min
Increase the dose of dopamine @ 15µg/kg/min at 30 min
Increase the dose of adrenaline @ 0.3µg/kg/min at 30 min
If goal not achieved after 15min
**RESCUE THERAPY: Treatment category A and C: inj, adrenaline @ 0.1µg/kg/min and titrate accordingly; Treatment category B: ↑the dose of inj. Adrenaline up to 0.5µg/kg/min (or consultant discretion). If goal not achieved, then start inj. nor-adrenaline @ 0.1µg/kg/min & then titrate accordingly

SPIRIT 2013 Checklist: Recommended items addressed in the clinical trial "Management of fluid refractory shock in children with SAM or severe underweight and diarrhea" protocol and related documents

**Section/ item**	**Item No**	**Description**	**Addressed on page number**
**Administrative information**			
Title	1	Descriptive title identifying the study design, population, interventions, and, if applicable, trial acronym	1
Trial registration	2a	Trial identifier and registry name. If not yet registered, name of intended registry	ClinicalTrials.gov, ID: NCT (page 2)
	2b	All items from the World Health Organization Trial Registration Data Set	-
Protocol version	3	Date and version identifier	2
Funding	4	Sources and types of financial, material, and other support	16
Roles and responsibilities	5a	Names, affiliations, and roles of protocol contributors	1, 16
	5b	Name and contact information for the trial sponsor	icddr,b
	5c	Role of study sponsor and funders, if any, in study design; collection, management, analysis, and interpretation of data; writing of the report; and the decision to submit the report for publication, including whether they will have ultimate authority over any of these activities	16
	5d	Composition, roles, and responsibilities of the coordinating centre, steering committee, endpoint adjudication committee, data management team, and other individuals or groups overseeing the trial, if applicable (see Item 21a for data monitoring committee)	IRB, icddr,b (Page 15)

For peer review only

**Introduction**			
Background and rationale	6a	Description of research question and justification for undertaking the trial, including summary of relevant studies (published and unpublished) examining benefits and harms for each intervention	2,3,4
	6b	Explanation for choice of comparators	2,3,4
Objectives	7	Specific objectives or hypotheses	4
Trial design	8	Description of trial design including type of trial (eg, parallel group, crossover, factorial, single group), allocation ratio, and framework (eg, superiority, equivalence, non-inferiority, exploratory)	4
**Methods: Participants, interventions, and outcomes**			
Study setting	9	Description of study settings (eg, community clinic, academic hospital) and list of countries where data will be collected. Reference to where list of study sites can be obtained	5
Eligibility criteria	10	Inclusion and exclusion criteria for participants. If applicable, eligibility criteria for study centres and individuals who will perform the interventions (e.g., surgeons, psychotherapists)	5
Interventions	11a	Interventions for each group with sufficient detail to allow replication, including how and when they will be administered	9, 10, 11
	11b	Criteria for discontinuing or modifying allocated interventions for a given trial participant (e.g., drug dose change in response to harms, participant request, or improving/worsening disease)	10, 11
	11c	Strategies to improve adherence to intervention protocols, and any procedures for monitoring adherence (e.g., drug tablet return, laboratory tests)	15
	11d	Relevant concomitant care and interventions that are permitted or prohibited during the trial	11

For peer review

Outcomes	12	Primary, secondary, and other outcomes, including the specific measurement variable (e.g., systolic blood pressure), analysis metric (e.g., change from baseline, final value, time to event), method of aggregation (e.g., median, proportion), and time point for each outcome. Explanation of the clinical relevance of chosen efficacy and harm outcomes is strongly recommended	12
Participant timeline	13	Time schedule of enrolment, interventions (including any run-ins and washouts), assessments, and visits for participants. A schematic diagram is highly recommended (see Figure)	6,7 (Table 1)
Sample size	14	Estimated number of participants needed to achieve study objectives and how it was determined, including clinical and statistical assumptions supporting any sample size calculations	12
Recruitment	15	Strategies for achieving adequate participant enrolment to reach target sample size	15
**Methods: Assignment of interventions (for controlled trials)**			
Allocation:			
Sequence generation	16a	Method of generating the allocation sequence (e.g., computer-generated random numbers), and list of any factors for stratification. To reduce predictability of a random sequence, details of any planned restriction (e.g., blocking) should be provided in a separate document that is unavailable to those who enrol participants or assign interventions	Permuted Block randomization, (page 5)
Allocation concealment mechanism	16b	Mechanism of implementing the allocation sequence (e.g., central telephone; sequentially numbered, opaque, sealed envelopes), describing any steps to conceal the sequence until interventions are assigned	Sequentially numbered, opaque, sealed envelopes (Page 5)

Implementation	16c	Who will generate the allocation sequence, who will enrol participants, and who will assign participants to interventions	Allocation sequence- Senior scientist not related to the study. Enrolment and assignment-PI & Study doctors (Page 5)
Blinding (masking)	17a	Who will be blinded after assignment to interventions (e.g., trial participants, care providers, outcome assessors, data analysts), and how	Open-labelled trial
	17b	If blinded, circumstances under which unblinding is permissible, and procedure for revealing a participant's allocated intervention during the trial	N/A
**Methods: Data collection, management, and analysis**			
Data collection methods	18a	Plans for assessment and collection of outcome, baseline, and other trial data, including any related processes to promote data quality (e.g., duplicate measurements, training of assessors) and a description of study instruments (e.g., questionnaires, laboratory tests) along with their reliability and validity, if known. Reference to where data collection forms can be found, if not in the protocol	7, 8, 9
	18b	Plans to promote participant retention and complete follow-up, including list of any outcome data to be collected for participants who discontinue or deviate from intervention protocols	15
Data management	19	Plans for data entry, coding, security, and storage, including any related processes to promote data quality (e.g., double data entry; range checks for data values). Reference to where details of data management procedures can be found, if not in the protocol	12, 13

Statistical methods	20a	Statistical methods for analysing primary and secondary outcomes. Reference to where other details of the statistical analysis plan can be found, if not in the protocol	12
	20b	Methods for any additional analyses (e.g., subgroup and adjusted analyses)	-
	20c	Definition of analysis population relating to protocol non-adherence (e.g., as randomised analysis), and any statistical methods to handle missing data (e.g., multiple imputation)	-
**Methods: Monitoring**			
Data monitoring	21a	Composition of data monitoring committee (DMC); summary of its role and reporting structure; statement of whether it is independent from the sponsor and competing interests; and reference to where further details about its charter can be found, if not in the protocol. Alternatively, an explanation of why a DMC is not needed	15
	21b	Description of any interim analyses and stopping guidelines, including who will have access to these interim results and make the final decision to terminate the trial	13
Harms	22	Plans for collecting, assessing, reporting, and managing solicited and spontaneously reported adverse events and other unintended effects of trial interventions or trial conduct	13
Auditing	23	Frequency and procedures for auditing trial conduct, if any, and whether the process will be independent from investigators and the sponsor	15
**Ethics and dissemination**			
Research ethics approval	24	Plans for seeking research ethics committee/institutional review board (REC/IRB) approval	15

Protocol amendments	25	Plans for communicating important protocol modifications (e.g., changes to eligibility criteria, outcomes, analyses) to relevant parties (e.g., investigators, REC/IRBs, trial participants, trial registries, journals, regulators)	15
Consent or assent	26a	Who will obtain informed consent or assent from potential trial participants or authorised surrogates, and how (see Item 32)	PI or his/her representative Study doctor (Page7)
	26b	Additional consent provisions for collection and use of participant data and biological specimens in ancillary studies, if applicable	N/A
Confidentiality	27	How personal information about potential and enrolled participants will be collected, shared, and maintained in order to protect confidentiality before, during, and after the trial	7
Declaration of interests	28	Financial and other competing interests for principal investigators for the overall trial and each study site	16
Access to data	29	Statement of who will have access to the final trial dataset, and disclosure of contractual agreements that limit such access for investigators	The PI will have the access to final data set
Ancillary and post-trial care	30	Provisions, if any, for ancillary and post-trial care, and for compensation to those who suffer harm from trial participation	N/A
Dissemination policy	31a	Plans for investigators and sponsor to communicate trial results to participants, healthcare professionals, the public, and other relevant groups (e.g., via publication, reporting in results databases, or other data sharing arrangements), including any publication restrictions	2,15
	31b	Authorship eligibility guidelines and any intended use of professional writers	NA

	31c	Plans, if any, for granting public access to the full protocol, participant-level dataset, and statistical code	
**Appendices**			
Informed consent materials	32	Model consent form and other related documentation given to participants and authorised surrogates	Attached
Biological specimens	33	Plans for collection, laboratory evaluation, and storage of biological specimens for genetic or molecular analysis in the current trial and for future use in ancillary studies, if applicable	7

For peer review only

BMJ Open

Efficacy of dopamine, adrenaline and blood transfusion for treatment of fluid refractory shock in children with severe acute malnutrition or severe underweight and cholera or other dehydrating diarrhoeas: protocol for a randomized controlled clinical trial

Journal:	*BMJ Open*
Manuscript ID	bmjopen-2022-068660.R2
Article Type:	Protocol
Date Submitted by the Author:	17-Mar-2023

Complete List of Authors:	Sarmin, Monira ; International Centre for Diarrhoeal Disease Research Bangladesh, Nutrition and Clinical Services Division<br>Shaly, Nusrat ; International Centre for Diarrhoeal Disease Research Bangladesh, Nutrition and Clinical Services Division<br>Sultana, Tania; International Centre for Diarrhoeal Disease Research Bangladesh, Nutrition and Clinical Services Division<br>Tariqujjaman, Md. ; International Centre for Diarrhoeal Disease Research Bangladesh<br>Shikha, Shamima ; International Centre for Diarrhoeal Disease Research Bangladesh, Nutrition and Clinical Services Division<br>Mariam, Nafisa; International Centre for Diarrhoeal Disease Research Bangladesh, Nutrition and Clinical Services Division<br>Jeorge, Didarul ; International Centre for Diarrhoeal Disease Research Bangladesh, Nutrition and Clinical Services Division<br>Tabassum, Mosharrat; International Centre for Diarrhoeal Disease Research Bangladesh, Nutrition and Clinical Services Division<br>Nahar, Baitun; International Centre for Diarrhoeal Disease Research Bangladesh<br>Afroze, Farzana; International Centre for Diarrhoeal Disease Research Bangladesh<br>Shahrin, Lubaba; International Centre for Diarrhoeal Disease Research Bangladesh, NCSD<br>Hossain, Md. Iqbal ; International Centre for Diarrhoeal Disease Research Bangladesh, Nutrition and Clinical Services Division<br>Alam, Baharul; International Centre for Diarrhoeal Disease Research Bangladesh, Nutrition and Clinical Services Division<br>Faruque, ASG; International Centre for Diarrhoeal Disease Research, Nutrition and Clinical Services Division<br>Islam, M; International Centre for Diarrhoeal Disease Research Bangladesh, Nutrition and Clinical Services Division<br>Osmany, Mohammad ; Bangabandhu Sheikh Mujib Medical University, Department of Cardiology<br>Chowdhury, Meshkat A.; Bangabandhu Sheikh Mujib Medical University, Department of Cardiology<br>Manji, Karim; Muhibili University of Health and Allied Sciences, Department of Pediatrics and Child Health<br>Kissoon, Niranjan; University of British Columbia, Department of

	Pediatrics<br>Chisti, Md.; International Centre for Diarrhoeal Disease Research Bangladesh, Nutrition and Clinical Services Division<br>Ahmed, Tahmeed; International Centre for Diarrhoeal Disease Research Bangladesh, Nutrition and Clinical Services Division
**Primary Subject Heading**:	Paediatrics
Secondary Subject Heading:	Gastroenterology and hepatology, Intensive care, Infectious diseases, Paediatrics
Keywords:	Paediatric intensive & critical care < ANAESTHETICS, Paediatric gastroenterology < PAEDIATRICS, Respiratory infections < THORACIC MEDICINE

SCHOLARONE™
Manuscripts

Title: Efficacy of dopamine, adrenaline and blood transfusion for treatment of fluid refractory shock in children with severe acute malnutrition or severe underweight and cholera or other dehydrating diarrhoeas: protocol for a randomized controlled clinical trial

Short Title: Efficacy of dopamine, adrenaline and blood transfusion for treatment of fluid refractory shock in severe malnutrition

For peer review only

Authors:

Monira Sarmin[1], Nusrat Jahan Shaly[1], Tania Sultana[1], Md. Tariqujjaman[1], Shamima Sharmin Shikha[1], Nafisa Mariam[1], Didarul Haque Jeorge[1], Mosharrat Tabassum[1], Baitun Nahar[1], Farzana Afroze[1], Lubaba Shahrin[1], Md. Iqbal Hossain[1], Baharul Alam[1], ASG Faruque[1], Md. Munirul Islam[1], Din-E-Mujahid Mohammad Faruqe Osmany[2], Chowdhury Meshkat Ahmed[2], Karim P Manji[3], Niranjan Kissoon[4], Mohammod Jobayer Chisti[1], Tahmeed Ahmed[1]

Affiliations:

[1]Nutrition and Clinical Services Division, icddr,b, Dhaka, Bangladesh

[2]Department of Cardiology, Bangabandhu Sheikh Mujib Medical University (BSMMU), Dhaka, Bangladesh

[3]Department of Paediatrics and Child Health, Muhimbili University of Health and Allied Sciences, Dar es Salaam, Tanzania

[4]Department of Paediatrics, University of British Colombia & British Colombia Children's Hospital, Canada

Corresponding author: Mohammod Jobayer Chisti (chisti@icddrb.org)

For peer review only - http://bmjopen.bmj.com/site/about/guidelines.xhtml

ABSTRACT

Introduction Diarrhoea is one of the leading causes of under-five childhood mortality and accounts for 8% of 5.4 million global under-5 deaths. In severely malnourished children, diarrhoea progresses to shock, where the risk of mortality is even higher. At icddr,b Dhaka Hospital, the fatality rate is as high as 69% in children with severe malnutrition and fluid refractory septic shock. To date, no study has evaluated systematically the effects of inotrope or vasopressor or blood transfusion in children with dehydrating diarrhoea (for example, in cholera) and severe acute malnutrition (SAM) or severe underweight who are in shock and unresponsive to WHO-recommended fluid therapy. To reduce the mortality of severely malnourished children presenting with diarrhea and fluid refractory shock, we aim to compare the efficacy of blood transfusion, dopamine, and adrenaline in fluid refractory shock in children who do not respond to WHO-recommended fluid resuscitation.

Methods and analysis In this randomized, three-arm, controlled, non-masked clinical trial in children 1-59 months old with SAM or severe underweight and fluid refractory shock, we will compare the efficacy of dopamine or adrenaline administration versus blood transfusion in children who failed to respond to WHO-recommended fluid resuscitation. The primary outcome variable is the case fatality rate. The effect of the intervention will be assessed by performing an intention-to-treat analysis. Recruitment and data collection began in July 2021 and are now ongoing. Results are expected by May 2023.

Ethics and dissemination This study has been approved by the icddr,b Institutional Review Board. We adhere to the 'Declaration of Helsinki' (2000), guidelines for Good Clinical Practice. Before enrolment, we are collecting signed informed consent from the parents or caregivers of the children. We will publish the results in a peer-reviewed journal and will arrange a dissemination seminar.

Trial registration number ClinicalTrials.gov**;** NCT04750070, February 11, 2021

Version no: 2.2, Dated July 21, 2022

For peer review only - http://bmjopen.bmj.com/site/about/guidelines.xhtml

STRENGTHS AND LIMITATIONS OF THIS STUDY

⇒This study includes severely malnourished children with diarrhoea having fluid refractory shock

⇒This study evaluates the cardiac function of the study children at the bedside by point-of-care cardiac ultrasound

⇒ This study is excluding children Down's syndrome or any chronic illness

⇒ Evaluation of non-diarrheal children having shock is out of scope for this study

For peer review only

INTRODUCTION

Background and rationale

Diarrheal diseases are the second leading cause of death in under-five children and were responsible for the deaths of 370,000 children in 2019 [1]. In Bangladesh, diarrhea causes 7.3% of under-5 deaths in 2019[2]. Children with severe acute malnutrition (SAM) frequently present with diarrhea, and fluid refractory shock is one of the common complications of diarrhea [3]. The management of these children is challenging. There is a lack of evidence-based guidelines for the management of fluid refractory shock in severely malnourished children. Optimized management of the altered hemodynamics in fluid refractory shock is yet to be explored and different opinions exist among experts, especially for malnourished children. Data from the Dhaka Hospital of icddr,b demonstrates that despite appropriate care, 69% of children having co-morbidities such as severe malnutrition die from fluid refractory shock [4].

The World Health Organization (WHO) recommends the administration of isotonic saline boluses in a dose of 15 mL/kg over 1 hour (maximum two times), followed by blood transfusion in a dose of 10 mL/kg over 3 hours in cases unresponsive to isotonic fluid boluses [5]. If blood pressure does not restore to the desired level after fluid bolus, the condition is termed as a fluid refractory/septic shock. Quick arrangement of safe blood is difficult, especially in resource-poor settings. The FEAST trial showed significantly higher mortality associated with the administration of saline bolus or albumin [6] in children having severe sepsis. Inotropes like dopamine perform better than excessive fluid administration [7] when myocardial dysfunction or vasoregulatory failure results in shock rather than absolute hypovolemia [7], [8]. Recent studies also demonstrated that adrenaline resulted in the early resolution of shock with survival benefits than dopamine [9], [10]. It is a complex situation whether to use blood or inotropes in

For peer review only - http://bmjopen.bmj.com/site/about/guidelines.xhtml

fluid refractory shock. Severely malnourished children are in reductive adaptation with different hemodynamics than well-nourished children. Despite the very high death rates, the management of fluid refractory shock in severely malnourished children with early inotropes or vasopressors has never been studied.

Malnourished children have less left ventricular mass [11], [12] as well as left ventricular function as evidenced by echocardiography [11], [13] which may constitute a bad prognostic parameter. The FEAST trial reported that in the bolus group, a significantly higher proportion had a terminal event cardiac rather than respiratory or neurological [14]. Point of care (POC) cardiac ultrasound is an emerging tool to assess cardiac function and fluid responsiveness in shock and it is non-invasive. Using the POC ultrasound to differentiate hypovolemic shock from distributive shock e.g. fluid refractory/septic shock, measuring ventricular function and IVC collapsibility can reduce the diagnostic time and increase the accuracy of diagnosis [15], [16]. Usually, in hypovolemic shock, both end-diastolic and end-systolic volumes are reduced [16], left ventricle (LV) is small having a hyperdynamic wall motion [17]. Whereas in fluid refractory/septic shock, LV is hyperdynamic [17] and only end-systolic volume is reduced [16]. Again, the diameter of the IVC at inspiration and expiration and their ratio provides useful information regarding fluid responsiveness [16]. To date, no study has evaluated systematically the effects of vasopressor and inotrope(s) in children with dehydrating diarrhea (for example, in cholera) and SAM having fluid refractory shock unresponsive to conventional WHO-recommended fluid therapy.

The proposed study will provide valuable data to determine whether resuscitation strategies including vasopressor and inotropes or blood result in increased survival in children who are severely malnourished with fluid refractory shock and diarrhea. In addition, POC cardiac ultrasound will provide information on whether the shock resulted from a fluid deficit or cardiac dysfunction.

For peer review only

Objective

The study aims to compare the efficacy of blood transfusion, dopamine, and adrenaline in fluid refractory shock in children having SAM or severe underweight who do not respond to WHO-recommended fluid resuscitation.

Trial design

This is a randomized, three-arm, controlled, non-masked clinical trial in children 1-59 months old with SAM or severely underweight and shock (Figure 1). With parental written informed consent, children are allocated to the study interventions following randomization, in addition to usual supportive care that includes broad-spectrum antibiotics, oxygen inhalation, IV glucose, etc. The study protocol follows the

For peer review only - http://bmjopen.bmj.com/site/about/guidelines.xhtml

Standard Protocol Items: Recommendations for Interventional Trials (SPIRIT) guidance for protocol reporting (modified to reflect the ongoing study activities) [18] (Table 1).

METHODS

Participants and interventions and outcomes

Study setting

The study is being conducted at the Dhaka Hospital of the icddr,b, Dhaka, Bangladesh. icddr,b is the largest diarrheal diseases hospital in the world. Annually a total of 194,838 patients of all ages and genders got treatment and care from icddr,b, and 57.2% were under five years of age. Besides diarrheal illness, many patients have other health conditions, like pneumonia, malnutrition, sepsis, and electrolyte abnormalities. For critically ill patients, there is an intensive care unit facility present within the hospital, equipped with contemporary life support measures, including mechanical ventilators, bubble continuous positive airway pressure (BCPAP) oxygen therapy, high flow nasal cannula (HNFC) oxygen therapy, and syringe pumps for vasopressor support. The ICU of the Dhaka Hospital of icddr,b manages around 1000 pediatric patients each year. More than 80% of them have a poor socio-economic background. About one-third have severe malnutrition, and one-fourth of them have severe sepsis or fluid refractory shock, there are other different wards for the treatment of diarrheal illness, respiratory problems, and malnutrition. icddr,b possesses a well-equipped laboratory service capable of performing a range of clinical tests proposed in this study.

Inclusion criteria are children aged 1-59 months of either sex having diarrhea and severe acute malnutrition who develop fluid refractory shock either at admission or at any point during their treatment in the hospital (which might be due to cholera or other dehydrating diarrhea or severe sepsis). We also included children having severe underweight as they are also vulnerable and their mortality rate is also high. We are using corrected weight for the anticipated loss of fluids to calculate severe acute malnutrition (SAM) or severe underweight.

Exclusion criteria are severe anaemia (hemoglobin < 5 gm/dl) and sclerema where blood transfusion is a mandate, congenital anomalies (tetralogy of Fallot (TOF)/atrial or ventricular septal defect (ASD or VSD)/Trisomy 21, etc.), negative blood groups, a requirement of cardio-pulmonary resuscitation during screening and non-consent from the caregivers/parents.

Randomization: The permuted block randomization technique is followed to select treatment arms for each child. The randomization procedure was planned and executed by a scientist from icddr,b who is not

For peer review only - http://bmjopen.bmj.com/site/about/guidelines.xhtml

involved in the study. The randomization list containing the subject identification numbers (IDs) and the corresponding group allocation remained concealed. IDs are chronologically assigned to each new study participant. After randomization, an opaque envelope containing the name of the allocated intervention group is opened.

Study duration: Twenty-four months

For peer review only

For peer review only - http://bmjopen.bmj.com/site/about/guidelines.xhtml

Table 1: Standard Protocol Items: Recommendation for Interventional Trials (SPIRIT) Figure. The schedule of enrolment, interventions, and assessments

**Indicators**		**Study period**						
	Screening	Enrolment & allocation	Study drugs administration & F-U	Rescue therapy	Hosp course up to disposal	Follow-up after discharge		
						1st follow up	2nd follow up	28th day follow-up (over phone)
Time point	Day 0	Hour 0	Hour 0-3 (1 hour for Dopamine & Adrenaline, 3 hours for Blood)	Hour 1- disposal (for Dopamine & Adrenaline) Hour 3- disposal (for Blood)		Day 7±2	Day 21±2	Day 28±3
Enrolment								
Eligibility screen	x							
Informed consent	x	x						
Randomization and allocation		x	X					
Interventions								
Different drugs (Dopamine or Adrenaline or Blood)			X					
Assessments:								
Demographic		x						
Comorbidities		x						
Physical examination	x	x	X	x	x	x	x	-
Primary outcome:								
case fatality								x
Secondary outcomes:								
Treatment failure			X					
MAP stabilization at 48 hours after enrolment				x	x			
Mechanical ventilation			X	x	x			
Incidence of heart failure			X	x	x			
Duration of ICU stay					x			
Duration of total hospitalization					x			
Time-to-achieve recovery from shock			X	x	x			
Ventricular function by cardiac USG	x	x		x	x (if require)			
**IVC (Inferior Vena Cava) collapsibility**	x	x		x	x (if require)			

For peer review only

For peer review only - http://bmjopen.bmj.com/site/about/guidelines.xhtml

[a]Detection of leptin from stored blood		x						

[a]According to the addendum (ERC approval on October 21, 2021), at the end of the study, stored blood samples will be tested for leptin.

Collection of Baseline Information

Any SAM children with fluid refractory shock, either at admission or developed at some point during their treatment period, within the defined age group are screened for study eligibility criteria by the study physician. According to the inclusion and exclusion criteria, parents or attending caregivers of those eligible children are invited for providing consent for their children to be enrolled in the study. A signed written informed consent form by parents or caregivers gives data concerning the study, attainable advantages and risks, and voluntary nature of participation, as well as the right to cancel the child's participation in the study at any time (even after giving the initial consent) while not providing any reason; after this, children are enrolled by the study physician. One copy of the signed consent document was given to the caregiver of every participant and another copy was reserved for the study documentation. A pretested case record form is used to collect relevant medical history information, including the nature and duration of illness, medication for current illness, and any history of significant past illness including pneumonia and diarrhea. The form is also used to collect information on sociodemographic characteristics, such as age, sex, religion, gestational age, parental age with education and occupations, source of drinking water and sanitation, type of fuel use, cooking place and smoking history, average monthly income of the family, number of siblings, and number of rooms in the home. Information is also collected about each child's feeding practice, which included their history of breastfeeding, formula feedings, or alternative complementary feedings, and also immunization status. Data on clinical examination findings are recorded by the study physician and include pulse, respiratory rate, axillary temperature, anthropometric measurements (ie, height, weight, mid-upper arm circumference for calculation of Z score), chest auscultation, oxygen saturation, presence or absence of chest wall indrawing, cyanosis, and mental status (ie, normal, irritable, or lethargic), and response according to AVPU. Each child's weight is measured by using an electronic weighing scale with a precision of 0.1 kg; length is measured using a standard-length board with a precision of 0.5 cm and mid-upper arm- circumference (MUAC) is measured using a standard MUAC tape with a precision of 0.2 cm by the trained and experienced study nurse of Dhaka hospital. Fever is defined when the axillary temperature is 38°C or greater. Respiratory rate is counted for a full 60 seconds by exposing the trunk when the child is awake and calm; the presence of lower-chest-wall indrawing is noted at the same time.

For peer review only - http://bmjopen.bmj.com/site/about/guidelines.xhtml

Oxygen saturation is measured using a portable pulse oximeter (Handheld Vital Signs Monitors- NT1D, Solaris Medical Technology, Inc, USA) with a probe on a finger or toe when the child breathes in room air. Oxygen saturation of <90% is defined as hypoxemia, which is an indication for supplemental oxygen therapy by using BCPAP. Blood group and Rh typing are done at the bedside and we exclude the child having negative blood groups.

Bedside Hb (if not known) is checked by using Hemocue analyser during the screening period to exclude severely anemic children requiring an urgent blood transfusion. The length of ICU stays, the total length of hospitalization and time-to-achieve-recovery (time required for the restoration of MAP after the starting of the intervention for fluid refractory shock) are recorded. We are also keeping a record of stool and urine volume. Any deterioration or adverse events (ie, signs of heart failure or need for mechanical ventilation) are also documented. Both the study and outcome are recorded including treatment failure/success, hospital discharge, referrals, and deaths or left against medical advice (LAMA).

Laboratory Testing and sample handling

We are performing laboratory tests after the enrolment of a child in the study that includes blood for haemoglobin percentage, total and differential counts of white blood cell (WBC), hematocrit, blood culture and antimicrobial susceptibility of pathogens, serum electrolytes and creatinine, calcium and magnesium, serum bilirubin, serum lactate, C-reactive protein (CRP), procalcitonin, and cardiac troponin I, stool (rectal swab for Cholera, Salmonella, Shigella and Campylobacter) and urine culture, chest radiograph, and arterial or capillary blood gas analysis. We are also doing a test for the exclusion of dengue during the dengue prevalent period in the country. We are checking serum lactate at enrolment and 4-6 hours after enrolment. Our target is to send samples for laboratory tests within 30 min-1 hour of enrolment. When a child is in shock, very often it is difficult to get an adequate amount of blood for proposed testing. Then we prioritize tests based on clinical relevance. We are doing the blood grouping, screening and cross-matching while we are following a child for study eligibility. We are performing cardiac ultrasonography during screening, enrolment and 4-6 hours later after enrolment. As we are enrolling children 24X7, the study physicians are doing the bedside cardiac ultrasound and storing the images of specific pre-selected parameters. Later the images are reviewed by experienced cardiologists to get the measurements.

We are archiving blood samples and planning to check serum leptin at the end of the study.

For peer review only - http://bmjopen.bmj.com/site/about/guidelines.xhtml

Study intervention and procedure

This is a three-arm trial where the intervention arms (dopamine and adrenaline) will be compared with the WHO standard intervention (blood transfusion). All study children stay at ICU for close monitoring. During the screening, children receive intravenous isotonic fluid @ of 15mL/kg over one hour (maximum two boluses i.e. up to 30ml/kg) as per WHO standard of care. The choice of isotonic fluid is Ringer`s lactate/Hartsol (Normal saline if any suspicion of hyperkelemia, acetate if severe dehydration is predominant). Dextrose is added if a child has hypoglycaemia with a random blood sugar <3 m mol/l. The study team members work to assess the eligibility and discuss with the parents/caregiver for possible enrolment. the regular ICU team helps in patient care. At the end of the screening, if the fluid refractory shock is diagnosed, then the study team proceed with written consent to avert the gap between consent to starting appropriate management of the child.

Children with fluid refractory shock are randomized to blood transfusion, dopamine, or adrenaline therapy. Children in the dopamine arm (**Treatment category A**) receive dopamine, 8 microgram/kg.min (increasing the dose after 15 minutes to 12 microgram/kg/min to a maximum of 15 microgram/kg/min, up to the achievement of goal). Children in the adrenaline arm (**Treatment category B**) receive adrenaline, 0.1 microgram/kg/min (increasing the dose after 15 minutes to 0.2 microgram/kg.min to a maximum of 0.3 microgram/kg.min, up to the achievement of goal). We are using peripheral lines to give dopamine or adrenaline. Drugs are prepared as IV infusion and instilled by an infusion/syringe pump. Children in the blood transfusion arm (**Treatment category C)** receive a transfusion of whole human blood in a dose of 10 mL/kg over 3 hours (Figure 1). To arrange blood within the time frame, we have an institutional volunteer donor list, two medical technologists dedicatedly work for this study, and we are also excluding children having negative blood groups. Even after that, If blood could not be arranged within one hour after the event of fluid refractory shock, we will start rescue therapy considering the event as treatment failure. In addition to the study interventions, broad-spectrum antibiotic therapy, oxygen therapy, and frequent monitoring, all children receive IV maintenance fluids with half-strength normal saline and 5% dextrose @ of 3 ml per kg per hour during the arrangement of blood transfusion and intervention with inotropes. Along with maintenance fluid, we add IV fluid if diarrhoea continues or a child develops dehydration. IV fluid maintenance is continued for 6 hours after the achievement of a goal and is further continued if the goal is not achieved. Our goal is to achieve MAP >50 mm of Hg and/or urine output ≥1 ml/kg per hour. After randomization, we monitor children at every 15-minute interval during the intervention to assess mental status by using AVPU, an acronym from "alert, verbal, pain, unresponsive", capillary refill time, rate and volume of the radial pulse, cold/warm extremities, urine output, blood pressure, respiratory rate, peripheral capillary O_2 saturation, and features of heart failure. We measure BP

For peer review only - http://bmjopen.bmj.com/site/about/guidelines.xhtml

manually using the standard cuff. To minimize the use of a catheter, we apply a paediatric urine collector bag and measure the urine output. However, for a female child, it is difficult to measure. We are considering an indwelling catheter if require. Mechanical ventilation is provided if the child did not respond to bubble CPAP (if SpO2<85% at least after an hour of bubble CPAP oxygen therapy) or had a respiratory failure (Ph<7.2 and PCO_2>60 mm of Hg in ABG analysis or SpO_2/FiO_2<265). Children who develop complications such as acute abdomen not responding to conservative management or acute renal insufficiency requiring dialysis are referred to a suitable higher facility for proper management. At the achievement of the goal, we continue the dose of dopamine or adrenaline for 6 hours. We follow up with the child every 30 min to 1-hour interval. Thereafter we reduce the dose in a stepwise manner similar to how we increased the dose. If the child becomes hypotensive again, we re-introduce the previous dose and follow the same procedure for further iteration.

Rescue therapy

The failure of any intervention is declared as a treatment failure. Then for children in the dopamine and blood transfusion arm, we start adrenaline as rescue therapy in a stepwise manner starting from 0.1 microgram/kg/min and increasing every 15 min by 0.1 microgram/kg/min up to restoration of the desired goal (MAP >50 mm of Hg or urine output>1ml/kg/hour) and the highest dose is 0.5 microgram/kg/min (or consultant discretion) For children in the adrenaline arm, the dose is increased every 15 minutes from 0.3 microgram/kg/min (the highest proposed intervention dose) to a max of 0.5 microgram/kg/min. Upon achieving the goal, we continue the same dose for 4-6 hours and then we de-escalate in a stepwise manner.

If the goal is not achieved with injection of adrenaline then injection of noradrenaline is started @ 0.1 microgram/kg/min and we increase the dose every 15 min by 0.1 microgram/kg up to restoration of the desired goal (MAP >50 mm of Hg or urine output>1ml/kg/hour) and the highest dose is 0.5 microgram/kg/min. Upon achieving the goal, we continue the same dose for 6 hours and then will de-escalate in a stepwise manner. For those patients whose MAP is not restored with inotropes, injection hydrocortisone @ 2mg/kg every 6 hours is added [19]. During rescue therapy, for doses and selection of any inotrope(s), we are following the recommendation from the PICU consultant as all cases are not the same.

Feeding and other care

Depending on the clinical condition either we keep the child NPO or start trophic feeding for children who had MAP >50 mm of Hg and had no evidence of paralytic ileus. Feeds consist of "Milk Suzi" (similar to WHO recommended F-75) for children older than 6 months, and modified infant formula (in

For peer review only - http://bmjopen.bmj.com/site/about/guidelines.xhtml

addition to breast milk, if applicable) is given to infants 1-6 months of age [20]. Thus, we provide a fluid volume of 120ml/kg/day to deliver 80-82 Kcal/kg/day. A child is kept warm to prevent hypothermia. If there is hypoglycaemia (random blood glucose < 3mmol/L), correction is given with 10% glucose intravenously in a dose of 5 mL/kg to be followed by 10% glucose administration through a nasogastric tube, following the standard protocol of the hospital.

Nutritional rehabilitation

As the study children have severe acute malnutrition, they require nutritional rehabilitation. After recovering from acute illness, the mothers/caregivers are asked to stay in the hospital's Nutritional Rehabilitation Unit (NRU) for 2-3 weeks. In the NRU, the standard dietary protocol is followed for nutritional rehabilitation and the children are discharged upon fulfilling discharge criteria.

Post-discharge follow-up

We follow up with a child on the 28th day (±3 days) of enrolment to know their clinical status over the phone. In addition, the children who were discharged from NRU or taking early discharge from the ward, are requested to come for a pre-scheduled follow-up at the hospital nutrition follow-up unit (NFU) of icddr,b. We followed the children at NFU and record their nutritional status and health problems. Our scheduled follow-ups for this study children are in two-time points; once after 7 days of discharge, then after 15 days of 1st follow-up. If any mother/caregiver fails to attend a follow-up visit, then we communicate with the mother/caregiver over the phone for two-time points. We document the outcome whether they are alive or not, and had any intercurrent illness e.g. diarrhea, pneumonia.

Outcome measures

Primary outcome variable is the case fatality rate (at 28±3 days from the day of enrolment). The secondary outcome variables are the rate of treatment failure, rate of MAP stabilization at 48 hours after enrolment, need for mechanical ventilation, the incidence of heart failure, duration of ICU stay and total hospitalization, time-to-achieve recovery, evaluation of cardiac function by point of care ultrasonogram (right ventricular function, left ventricular function and Inferior Vena Cava (IVC) collapsibility).

For peer review only - http://bmjopen.bmj.com/site/about/guidelines.xhtml

Sample size

The 2010-11 data from the ICU of the Dhaka Hospital of the icddr,b (the proposed study site) revealed that among the 36 severely malnourished children with shock, 22 died i.e. mortality rate among SAM children with septic shock was 61% (22/36). We assumed that the proposed interventions would reduce the case fatality rate by 50%, i.e. the rate will decrease from 61% to 31%.

To detect this magnitude of difference in the death rate, with 80% power (20% type II error) and 0.05 type I error, the required sample size is 40 children in each of the three groups. Considering around 10% dropouts after enrolment, the total sample size would be 45 children at least in each of the three groups, i.e. a total of 135 children - the final sample size of our study.

Data collection, management and analysis

Data will be collected using a hard copy structured case record form. Data will be entered into a personal computer on the online platform named Research Electronic Data Capture (REDCap). The data management team will check the consistency of data and will download and store the dataset as a backup to ensure data security. Statistical analyses will include descriptive as well as analytical methods. The effect of the intervention will be assessed by performing an intention-to-treat analysis as well as a per-protocol analysis. We anticipate that there will be no differences in the socio-demographic data among the three groups. Categorical variables will be compared using the Chi-square test or Fisher's exact test as appropriate. When the variables of interest are continuous and distributed normally, the statistical significance of group means will be evaluated by analysis of variance. When the variables are continuous and not normally distributed, the statistical significance of group differences will be determined by the Kruskal-Wallis test. The post hoc test will be done if required. To determine the differences in primary outcome i.e. case fatality among groups, we will perform the Chi-square test or Fisher's exact test as appropriate. The multiple generalized linear model (log-binomial regression) will be performed to explore the effect of interventions on case fatality after adjusting for potential confounders including the child's age, child's sex, child's morbidity status, wealth status, etc. The strength of the differences will be determined by calculating the relative risks (RRs) with 95% confidence intervals (CIs). In addition, we will analyze the Kaplan-Meier survival curve to compare the survival times among the three treatment groups. A probability value of less than 0.05 will be considered statistically significant. The scheduled interim analysis will be done after 50% of enrolment or earlier if the data safety and monitoring board (DSMB) advise. DSMB is an independent body, they review and follow the entire study and provide time-to-time suggestions. We will follow the stopping rule if the $P < 0.003$, as recommended by Pato and

For peer review only - http://bmjopen.bmj.com/site/about/guidelines.xhtml

Fleming [21]. Data analysis will be done using SPSS for Windows (Version 20.0. Armonk, NY: IBM Corp and Epi Info (version 7.0, USD, Stone Mountain, GA) or STATA (version 15.0 SE).

Reporting of adverse events

Serious adverse events (deaths) after initiation of the study intervention are recorded and presented to the DSMB within 24 hours of the event. The report includes a detailed clinical history, the study intervention, and management given until the outcome. We contacted over the phone for study participants who are referred.

Operational definition

Severe Acute Malnutrition (SAM): A child with a <-3 Z-score of weight for height/length (WHZ/WLZ) of the median value of the WHO standard or MUAC<115 mm (for children >6 months of age) /visible wasting (for children <6 months of age), or presence of nutritional edema.

Severe underweight: A child with a <-3 Z-score of weight for age (WAZ) of the median value of the WHO standard.

Sepsis: Presence of infection/inflammation plus hyperthermia or hypothermia (temperature >38.5º Celsius or <35.0º Celsius respectively) plus tachycardia (HR: infant>160/min, 1-5 years>140/min) or abnormal WBC count ($>12\times10^9/L$* or, $<4\times10^9/L$ or, band and neutrophil ratio ≥ 0.1).

Severe sepsis: Severe sepsis is defined as the presence of sepsis plus organ dysfunction or tissue hypoperfusion [poor peripheral perfusion (hypotension and/ or absent peripheral pulses and/or delayed capillary refilling time (CRT) in the absence of dehydration or after correction of dehydration)].

Fluid refractory shock or Septic Shock: Shock is defined as hypotension (Mean arterial pressure <50mm of Hg) and or reduced urine output <1 mL/kg/h despite fluid resuscitation [22]. This 50 mm Hg cut-off was fixed on the basis of our local evidence from the same population [22]. A child in septic shock usually has a cold periphery, delayed CRT, low volume or absent pulse.

Mean arterial pressure (MAP): MAP is calculated from systolic blood pressure (SBP) and diastolic blood pressure (DBP) by using a formula: DBP added to 1/3rd of pulse pressure (subtraction of the DBP from the SBP).

Treatment failure: Treatment failure is defined if an intervention results in

- ☐ MAP < 50 mm of Hg or
- ☐ Urine output is < 1 ml/kg per hour, at the end of intended intervention over 2/3 hour

For peer review only - http://bmjopen.bmj.com/site/about/guidelines.xhtml

For peer review only

Heart failure (a combination of findings)

- ☐ Age-specific tachypnea
- ☐ Tachycardia
- ☐ Enlarged tender liver
- ☐ Pedal edema (new or worsening)
- ☐ Basal crackles and/or
- ☐ Gallop and
- ☐ Response to furosemide

Signs of improvement (during 1st hour/2nd hour of fluid bolus therapy)

- ☐ Pulse rate falls
- ☐ Pulse volume improves
- ☐ Respiratory rate falls
- ☐ No signs of pulmonary edema

Signs of deterioration (during 1st hour/2nd hour of fluid bolus therapy)

- ☐ Breathing rate increases by 5/minute
- ☐ Pulse rate increases by 15/minute
- ☐ Liver enlarges (by measuring size at the beginning of fluid resuscitation and at the time of deterioration)
- ☐ Fine crackles at the lung base or throughout the lung fields
- ☐ Edema (new or worsening)
- ☐ Galloping heart rhythm

Acute kidney injury (AKI): AKI is defined as an increase in serum creatinine by ≥ 0.3 mg/dL from baseline within 48 hours OR, an increase in serum creatinine to ≥ 1.5 times baseline or age-specific upper limit of reference interval (ULRI) OR, urine volume ≤ 0.5 mL/kg/hour for six hours.

Recovery time: Time required for the restoration of MAP after starting the intervention for shock.

Congenital heart diseases: Atrial septal defect, ventricular septal defect, Tetralogy of Fallot (TOF).

Organic/other diseases: Down`s syndrome, cerebral palsy.

Patient and public involvement

We have planned to conduct public engagement activities like round table discussions and workshops at the end of the study.

For peer review only - http://bmjopen.bmj.com/site/about/guidelines.xhtml

Ethics

This study has been approved by the Institutional Review Board (IRB) of the icddr,b (Protocol No. PR#20021, version 2.2, dated 21.07.2022). We are conducting the study in compliance with the 'Declaration of Helsinki' (2000), Guideline for Good Clinical Practice (GCP). Before enrolment, we are collecting signed informed consent from the parents or caregivers of the children. We are strictly maintaining privacy, anonymity, and confidentiality of data or information identifying the patients or caregivers. Participants' medical information, particulars of treatment, and laboratory test results will be kept confidential and under lock and key; only the research staff will have access to this information. The risk to enrolled subjects from participation in the study is minimal. Adverse medical events are anticipated as the study is enrolling critically ill patients. This protocol has been reviewed & approved by the DSMB of icddr,b. DSMB is a five-membered board that independently reviews the activity of the study. We are reporting the occurrence of any serious adverse event (e.g., death) to the DSMB within 24 hours of the event and will follow their recommendations. There is no influence of the funder at any stage of the research, starting from study design and up to publication. Study patients are recruited into three different groups. For this clinical trial, inj adrenaline and inj dopamine are being used in fluid refractory shock. These drugs are already in use in the ICU of Dhaka hospital of icddr,b [22]. WHO recommends blood transfusion, and we are routinely practicing it. Regardless of the study result, finally, it will be disseminated.

Strategies for achieving adequate participant enrolment

Treatment at icddr,b Dhaka Hospital is free of cost for all patients. In addition to routine care, the study children get more follow-up by study physicians. So, they get the benefit of early detection and early intervention for any clinical condition. The caregiver has access to the study investigators and other physicians to communicate and to get an answer to their questions related to their child's medical condition and treatment even after their discharge. However, we pay a small amount of money (transport cost) for their follow-up visit after their usual discharge. We believe this information and empathetic care from the study will ensure adequate participant enrolment to reach the target sample size and also reduce the number of lost to follow-up.

Dissemination

After approval from the investigators of the protocol, we will publish the results and other findings of the study. We will use The International Committee of Medical Journal Editors guidelines to ascertain the authorship of papers.

For peer review only - http://bmjopen.bmj.com/site/about/guidelines.xhtml

Study status: As of August 2022, we have screened 49 children and enrolled 18 participants. Participants' screening and enrolment are ongoing.

Protocol amendments

After the first approval of the protocol, we included two cardiologists as co-investigators. After one year of enrolment, we made a change in the inclusion criteria also. Now we are including severely underweight children along with SAM children. So, we required amendments to the protocol which were all reviewed and approved by the icddr,b IRB. Now we are using version 2.2 of the protocol.

Contributorship statement

Monira Sarmin (MS), Nusrat Jahan Shaly (NJS), Tania Sultana (TS), Md Tariqujjaman (MT), Shamima Sharmin Shikha (SSS), Nafisa Mariam (NM), Didarul Haque Jeorge (DHJ), Mosharrat Tabassum (MT), Baitun Nahar (BN), Farzana Afroze (FA), Lubaba Shahrin (LS), Md. Iqbal Hossain (MIH), Baharul Alam (BA), ASG Faruque (ASGF), Md. Munirul Islam (MMI), Din-E-Mujahid Mohammad Faruqe Osmany (DEMMFO), Chowdhury Meshkat Ahmed (CMA), Karim P Manji (KPM), Niranjan Kissoon (NK), Mohammod Jobayer Chisti (MJC), Tahmeed Ahmed (TA).

The study concept and design were conceived by MS, BN, LS, FA, MIH, BA, ASGF, MMI, DEMMFO, CMA, KPM, NK, MJC, and TA. NJS, TA, FA, LS, SSS, NM, DHJ, MT, MJC, and MS are conducting patient screening, patient management, and data collection. Data management is performed by MT, NJS, TA, FA, SSS, NM, DHJ, MT, and MS. Analysis will be performed by MT, MS, MJC, LS, and TA. MS, LS, NJS, TS, MT and MJC prepared the first draft of the manuscript. All authors reviewed, edited and critiqued the manuscript.

Competing interest

None declared

Funding

This protocol is supported by Wellcome Trust, Grant number- 215691/Z/19/Z.

Acknowledgement

For peer review only - http://bmjopen.bmj.com/site/about/guidelines.xhtml

For peer review only

icddr,b is grateful to the governments of Bangladesh, Canada, Sweden and the UK for providing core/unrestricted support.

References

1. WHO. Diarrhoea. 2022

2. Unicef. Diarrhoea 2022 [Available from: https://data.unicef.org/topic/child-health/diarrhoeal-disease accessed 26 April 2022].

3. Chisti MJ, Pietroni MA, Smith JH, et al. Predictors of death in under-five children with diarrhoea admitted to a critical care ward in an urban hospital in Bangladesh. *Acta paediatrica* 2011;100(12):e275-e79.

4. Chisti MJ, Salam MA, Bardhan PK, et al. Severe sepsis in severely malnourished young Bangladeshi children with pneumonia: A retrospective case control study. *PloS one* 2015;10(10):e0139966.

5. Organization WH. Pocket book of hospital care for children: guidelines for the management of common childhood illnesses: World Health Organization 2013.

6. Maitland K, Kiguli S, Opoka RO, et al. Mortality after fluid bolus in African children with severe infection. *New England Journal of Medicine* 2011;364(26):2483-95.

7. Brierley J, Carcillo JA, Choong K, et al. Clinical practice parameters for hemodynamic support of pediatric and neonatal septic shock: 2007 update from the American College of Critical Care Medicine. *Critical care medicine* 2009;37(2):666.

8. Gill A, Weindling A. Randomised controlled trial of plasma protein fraction versus dopamine in hypotensive very low birthweight infants. *Archives of disease in childhood* 1993;69(3 Spec No):284-87.

9. Ventura AM, Shieh HH, Bousso A, et al. Double-Blind Prospective Randomized Controlled Trial of Dopamine Versus Epinephrine as First-Line Vasoactive Drugs in Pediatric Septic Shock. *Critical care medicine* 2015;43(11):2292-302. doi: 10.1097/ccm.0000000000001260 [published Online First: 2015/09/01]

10. Ramaswamy KN, Singhi S, Jayashree M, et al. Double-Blind Randomized Clinical Trial Comparing Dopamine and Epinephrine in Pediatric Fluid-Refractory Hypotensive Septic Shock. *Pediatr Crit Care Med* 2016;17(11):e502-e12. doi: 10.1097/pcc.0000000000000954 [published Online First: 2016/09/28]

11. ABUFADDAN N, Khalid E, Shams H, et al. Myocardial dysfunction in malnourished children. *Journal of Pediatric Sciences* 2010;2(2)

12. Olivares JL, Vazquez M, Rodríguez G, et al. Electrocardiographic and echocardiographic findings in malnourished children. *Journal of the American College of Nutrition* 2005;24(1):38-43.

13. El-Sayed H, Nassar M, Habib N, et al. Structural and functional affection of the heart in protein energy malnutrition patients on admission and after nutritional recovery. *European journal of clinical nutrition* 2006;60(4):502.

14. Maitland K, George EC, Evans JA, et al. Exploring mechanisms of excess mortality with early fluid resuscitation: insights from the FEAST trial. *BMC Med* 2013;11:68. doi: 10.1186/1741-7015-11-68 [published Online First: 2013/03/19]

For peer review only - http://bmjopen.bmj.com/site/about/guidelines.xhtml

15. Jones AE, Tayal VS, Sullivan DM, et al. Randomized, controlled trial of immediate versus delayed goal-directed ultrasound to identify the cause of nontraumatic hypotension in emergency department patients. *Critical care medicine* 2004;32(8):1703-8. doi: 10.1097/01.ccm.0000133017.34137.82 [published Online First: 2004/08/03]
16. Sun J-T. New Advances in Emergency Ultrasound Protocols for Shock. *J Med Ultrasound* 2017;25(4):191-94. doi: 10.1016/j.jmu.2017.09.005 [published Online First: 12/06]
17. Atkinson PRT, McAuley DJ, Kendall RJ, et al. Abdominal and Cardiac Evaluation with Sonography in Shock (ACES): an approach by emergency physicians for the use of ultrasound in patients with undifferentiated hypotension. *Emergency Medicine Journal* 2009;26(2):87-91. doi: 10.1136/emj.2007.056242
18. Chan A, Tetzlaff J, Altman D, et al. Krle a-Jeri K, et al. SPIRIT 2013 statement: defining standard protocol items for clinical trials. *Annals of Internal Medicine* 2013;158(3):200-7.
19. Weiss SL, Peters MJ, Alhazzani W, et al. Surviving sepsis campaign international guidelines for the management of septic shock and sepsis-associated organ dysfunction in children. *Intensive care medicine* 2020;46(1):10-67.
20. Ahmed T, Ali M, Ullah MM, et al. Mortality in severely malnourished children with diarrhoea and use of a standardised management protocol. *The Lancet* 1999;353(9168):1919-22.
21. Science PWCo. Frequentist Methods: O'Brien-Fleming, Pocock, Haybittle-Peto [Available from: https://online.stat.psu.edu/stat509/node/80/.

22.Sarmin M, Ahmed T, Bardhan PK, et al. Specialist hospital study shows that septic shock and drowsiness predict mortality in children under five with diarrhoea. *Acta Paediatr* 2014;103(7):e306-11. doi: 10.1111/apa.12640 [published Online First: 2014/03/26]

Figure 1

Study flow chart showing the procedures of screening and enrolment and allocation of a child in any of the three treatment categories and ways of rescue therapy if the study goal is not achieved

For peer review only

For peer review only - http://bmjopen.bmj.com/site/about/guidelines.xhtml

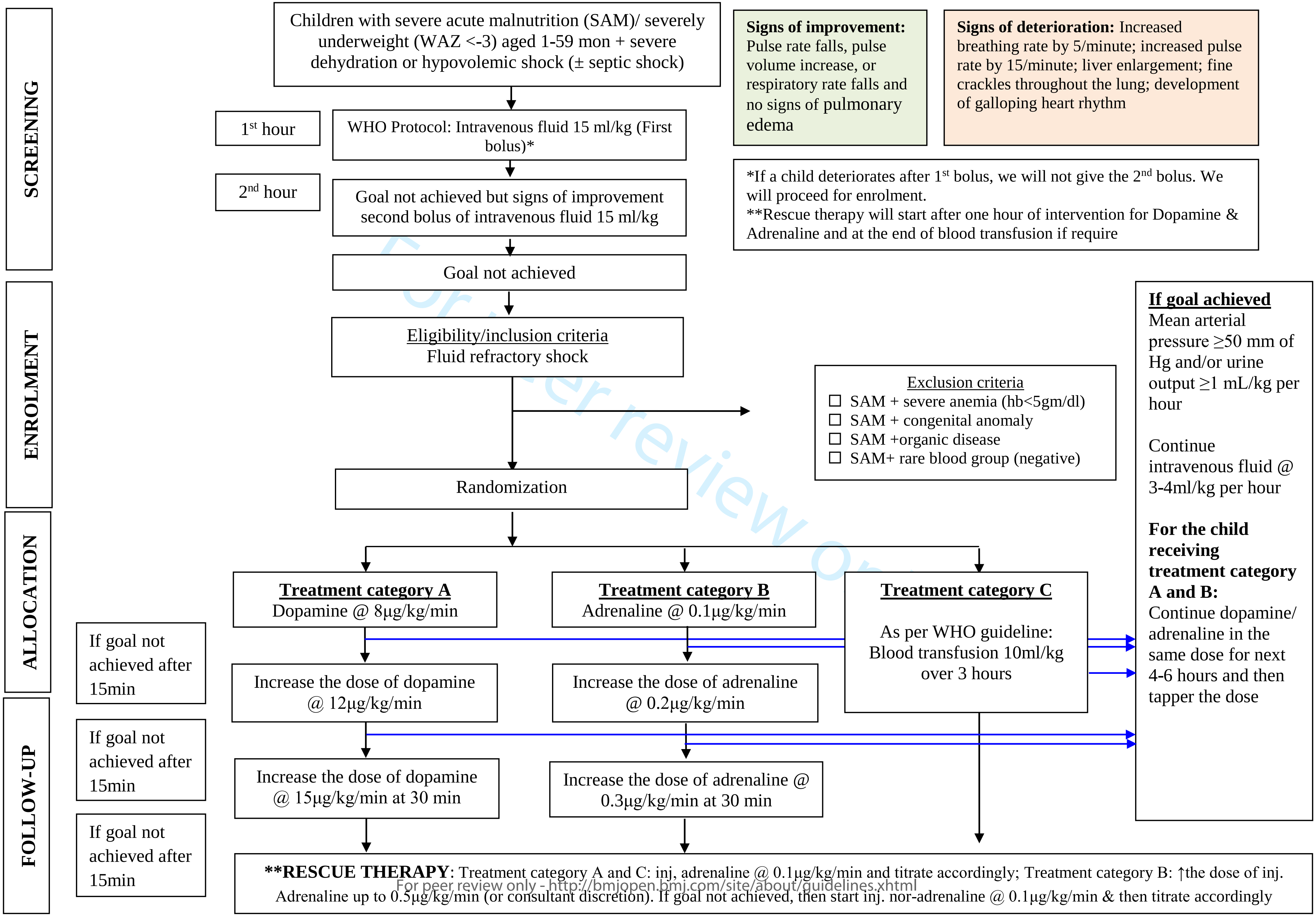

For peer review only - http://bmjopen.bmj.com/site/about/guidelines.xhtml

SPIRIT 2013 Checklist: Recommended items addressed in the clinical trial "Management of fluid refractory shock in children with SAM or severe underweight and diarrhea" protocol and related documents

Section/ item	Item No	Description	Addressed on page number
**Administrative information**			
Title	1	Descriptive title identifying the study design, population, interventions, and, if applicable, trial acronym	1
Trial registration	2a	Trial identifier and registry name. If not yet registered, name of intended registry	ClinicalTrials.gov, ID: NCT (page 2)
	2b	All items from the World Health Organization Trial Registration Data Set	-
Protocol version	3	Date and version identifier	2
Funding	4	Sources and types of financial, material, and other support	16
Roles and responsibilities	5a	Names, affiliations, and roles of protocol contributors	1, 16
	5b	Name and contact information for the trial sponsor	icddr,b
	5c	Role of study sponsor and funders, if any, in study design; collection, management, analysis, and interpretation of data; writing of the report; and the decision to submit the report for publication, including whether they will have ultimate authority over any of these activities	16
	5d	Composition, roles, and responsibilities of the coordinating centre, steering committee, endpoint adjudication committee, data management team, and other individuals or groups overseeing the trial, if applicable (see Item 21a for data monitoring committee)	IRB, icddr,b (Page 15)

For peer review only

**Introduction**			
Background and rationale	6a	Description of research question and justification for undertaking the trial, including summary of relevant studies (published and unpublished) examining benefits and harms for each intervention	2,3,4
	6b	Explanation for choice of comparators	2,3,4
Objectives	7	Specific objectives or hypotheses	4
Trial design	8	Description of trial design including type of trial (eg, parallel group, crossover, factorial, single group), allocation ratio, and framework (eg, superiority, equivalence, non-inferiority, exploratory)	4
**Methods: Participants, interventions, and outcomes**			
Study setting	9	Description of study settings (eg, community clinic, academic hospital) and list of countries where data will be collected. Reference to where list of study sites can be obtained	5
Eligibility criteria	10	Inclusion and exclusion criteria for participants. If applicable, eligibility criteria for study centres and individuals who will perform the interventions (e.g., surgeons, psychotherapists)	5
Interventions	11a	Interventions for each group with sufficient detail to allow replication, including how and when they will be administered	9, 10, 11
	11b	Criteria for discontinuing or modifying allocated interventions for a given trial participant (e.g., drug dose change in response to harms, participant request, or improving/worsening disease)	10, 11
	11c	Strategies to improve adherence to intervention protocols, and any procedures for monitoring adherence (e.g., drug tablet return, laboratory tests)	15
	11d	Relevant concomitant care and interventions that are permitted or prohibited during the trial	11

Outcomes	12	Primary, secondary, and other outcomes, including the specific measurement variable (e.g., systolic blood pressure), analysis metric (e.g., change from baseline, final value, time to event), method of aggregation (e.g., median, proportion), and time point for each outcome. Explanation of the clinical relevance of chosen efficacy and harm outcomes is strongly recommended	12
Participant timeline	13	Time schedule of enrolment, interventions (including any run-ins and washouts), assessments, and visits for participants. A schematic diagram is highly recommended (see Figure)	6,7 (Table 1)
Sample size	14	Estimated number of participants needed to achieve study objectives and how it was determined, including clinical and statistical assumptions supporting any sample size calculations	12
Recruitment	15	Strategies for achieving adequate participant enrolment to reach target sample size	15
**Methods: Assignment of interventions (for controlled trials)**			
Allocation:			
Sequence generation	16a	Method of generating the allocation sequence (e.g., computer-generated random numbers), and list of any factors for stratification. To reduce predictability of a random sequence, details of any planned restriction (e.g., blocking) should be provided in a separate document that is unavailable to those who enrol participants or assign interventions	Permuted Block randomization, (page 5)
Allocation concealment mechanism	16b	Mechanism of implementing the allocation sequence (e.g., central telephone; sequentially numbered, opaque, sealed envelopes), describing any steps to conceal the sequence until interventions are assigned	Sequentially numbered, opaque, sealed envelopes (Page 5)

Implementation	16c	Who will generate the allocation sequence, who will enrol participants, and who will assign participants to interventions	Allocation sequence- Senior scientist not related to the study. Enrolment and assignment-PI & Study doctors (Page 5)
Blinding (masking)	17a	Who will be blinded after assignment to interventions (e.g., trial participants, care providers, outcome assessors, data analysts), and how	Open-labelled trial
	17b	If blinded, circumstances under which unblinding is permissible, and procedure for revealing a participant's allocated intervention during the trial	N/A
**Methods: Data collection, management, and analysis**			
Data collection methods	18a	Plans for assessment and collection of outcome, baseline, and other trial data, including any related processes to promote data quality (e.g., duplicate measurements, training of assessors) and a description of study instruments (e.g., questionnaires, laboratory tests) along with their reliability and validity, if known. Reference to where data collection forms can be found, if not in the protocol	7, 8, 9
	18b	Plans to promote participant retention and complete follow-up, including list of any outcome data to be collected for participants who discontinue or deviate from intervention protocols	15
Data management	19	Plans for data entry, coding, security, and storage, including any related processes to promote data quality (e.g., double data entry; range checks for data values). Reference to where details of data management procedures can be found, if not in the protocol	12, 13

Statistical methods	20a	Statistical methods for analysing primary and secondary outcomes. Reference to where other details of the statistical analysis plan can be found, if not in the protocol	12
	20b	Methods for any additional analyses (e.g., subgroup and adjusted analyses)	-
	20c	Definition of analysis population relating to protocol non-adherence (e.g., as randomised analysis), and any statistical methods to handle missing data (e.g., multiple imputation)	-
**Methods: Monitoring**			
Data monitoring	21a	Composition of data monitoring committee (DMC); summary of its role and reporting structure; statement of whether it is independent from the sponsor and competing interests; and reference to where further details about its charter can be found, if not in the protocol. Alternatively, an explanation of why a DMC is not needed	15
	21b	Description of any interim analyses and stopping guidelines, including who will have access to these interim results and make the final decision to terminate the trial	13
Harms	22	Plans for collecting, assessing, reporting, and managing solicited and spontaneously reported adverse events and other unintended effects of trial interventions or trial conduct	13
Auditing	23	Frequency and procedures for auditing trial conduct, if any, and whether the process will be independent from investigators and the sponsor	15
**Ethics and dissemination**			
Research ethics approval	24	Plans for seeking research ethics committee/institutional review board (REC/IRB) approval	15

Protocol amendments	25	Plans for communicating important protocol modifications (e.g., changes to eligibility criteria, outcomes, analyses) to relevant parties (e.g., investigators, REC/IRBs, trial participants, trial registries, journals, regulators)	15
Consent or assent	26a	Who will obtain informed consent or assent from potential trial participants or authorised surrogates, and how (see Item 32)	PI or his/her representative Study doctor (Page7)
	26b	Additional consent provisions for collection and use of participant data and biological specimens in ancillary studies, if applicable	N/A
Confidentiality	27	How personal information about potential and enrolled participants will be collected, shared, and maintained in order to protect confidentiality before, during, and after the trial	7
Declaration of interests	28	Financial and other competing interests for principal investigators for the overall trial and each study site	16
Access to data	29	Statement of who will have access to the final trial dataset, and disclosure of contractual agreements that limit such access for investigators	The PI will have the access to final data set
Ancillary and post-trial care	30	Provisions, if any, for ancillary and post-trial care, and for compensation to those who suffer harm from trial participation	N/A
Dissemination policy	31a	Plans for investigators and sponsor to communicate trial results to participants, healthcare professionals, the public, and other relevant groups (e.g., via publication, reporting in results databases, or other data sharing arrangements), including any publication restrictions	2,15
	31b	Authorship eligibility guidelines and any intended use of professional writers	NA

For peer review only

	31c	Plans, if any, for granting public access to the full protocol, participant-level dataset, and statistical code	
**Appendices**			
Informed consent materials	32	Model consent form and other related documentation given to participants and authorised surrogates	Attached
Biological specimens	33	Plans for collection, laboratory evaluation, and storage of biological specimens for genetic or molecular analysis in the current trial and for future use in ancillary studies, if applicable	7

For peer review only
